# The effect and safety of constraint-induced movement therapy for post-stroke motor dysfunction: a meta-analysis and trial sequential analysis

**DOI:** 10.3389/fneur.2023.1137320

**Published:** 2023-04-18

**Authors:** Jiaming Zhang, Xianjun Xiao, Qizu Jin, Juan Li, Dongling Zhong, Yuxi Li, Yan Qin, Hong Zhang, Xiaobo Liu, Chen Xue, Zhong Zheng, Rongjiang Jin

**Affiliations:** ^1^School of Health Preservation and Rehabilitation, Chengdu University of Traditional Chinese Medicine, Chengdu, Sichuan, China; ^2^The Third Hospital of Mianyang, Sichuan Mental Health Center, Mianyang, Sichuan, China; ^3^Center for Neurobiological Detection, West China Hospital of Sichuan University, Chengdu, Sichuan, China

**Keywords:** constraint-induced movement therapy, motor dysfunction, stroke, trial sequential analysis, meta-analysis

## Abstract

**Background:**

Due to motor function insufficiency, patients with post-stroke motor dysfunction (PSMD) have limitations in performing an activity, feel restricted during social participation, and feel impaired in their quality of life. Constraint-induced movement therapy (CIMT) is a neurorehabilitation technique, but its effectiveness on PSMD after stroke still remains controversial.

**Objective:**

This meta-analysis and trial sequential analysis (TSA) aimed to comprehensively evaluate the effect and safety of CIMT for PSMD.

**Methods:**

Four electronic databases were searched from their inception to 1 January 2023 to identify randomized controlled trials (RCTs) investigating the effectiveness of CIMT for PSMD. Two reviewers independently extracted the data and assessed the risk of bias and reporting quality. The primary outcome was a motor activity log for the amount of use (MAL-AOU) and the quality of movement (MAL-QOM). RevMan 5.4, Statistical Package for Social Sciences (SPSS) 25.0, and STATA 13.0 software were used for statistical analysis. The certainty of the evidence was appraised using the Grading of Recommendations, Assessment, Development, and Evaluation (GRADE) system. We also performed the TSA to assess the reliability of the evidence.

**Results:**

A total of 44 eligible RCTs were included. Our results showed that CIMT combined with conventional rehabilitation (CR) was superior to CR in improving MAL-AOU and MAL-QOM scores. The results of TSA indicated that the above evidence was reliable. Subgroup analysis demonstrated that CIMT (≥6 h per day or duration ≤ 20 days) combined with CR was more effective than CR. Meanwhile, both CIMT and modified CIMT (mCIMT) combined with CR were more efficient than CR at all stages of stroke. No severe CIMT-related adverse events occurred.

**Conclusion:**

CIMT may be an optional and safe rehabilitation therapy to improve PSMD. However, due to limited studies, the optimal protocol of CIMT for PSMD was undetermined, and more RCTs are required for further exploration.

**Clinical trial registration:**

https://www.crd.york.ac.uk/PROSPERO/display_record.php?RecordID=143490, identifier: CRD42019143490.

## 1. Introduction

Motor dysfunction is one of the most common complications after stroke with a prevalence of up to 75–80% (1). Post-stroke motor dysfunction (PSMD) manifests as the insufficiency of motor function, including bradykinesia, ataxia, and muscle weakness, which have a negative impact on the quality of life in patients with stroke (2). It has been reported that over 80% of family members have to reduce working hours or even stop working to take care of patients with PSMD (3). PSMD imposes a heavy financial burden on individuals and families (4). Restoration of PSMD is one of the major goals in post-stroke rehabilitation.

Generally, due to pain, aversion, or repeated failure from previous attempts, patients with stroke may progressively avoid using the affected limbs in favor of the unaffected limbs (5). According to a review involving 66 studies, 55-85% of patients with PSMD prefer to complete activities of daily living with their unaffected limbs (6). Therefore, a learned non-use phenomenon gradually forms, which may hinder reorganization in the cortical representation of the affected limbs after stroke or slow down the recovery of coordinated bilateral movements related to daily activities (7).

Current rehabilitation modalities, such as physiotherapy (PT) and occupational therapy (OT), emphasize the use of the unaffected limbs to mobilize the affected limbs (8, 9). Although these rehabilitation modalities can achieve efficacy to some extent, patients with stroke may become reluctant to use the affected limbs, which can aggravate PSMD.

To address this problem, Taub and his colleagues proposed constraint-induced movement therapy (CIMT) (10). CIMT is one of the most developed neurorehabilitation techniques for motor restoration and is based on a theory of brain plasticity and cortical functional reorganization (11). Constraints and repeated practice may correct the learned non-use phenomenon and then improve the motor dysfunction of the affected limb (12). As a result, the use of the affected limbs may be increased, thus inducing a long-lasting improvement in PSMD.

Meta-analysis is an important way to synthesize existing studies. However, as evidence accumulates, an increasing frequency of statistical tests in meta-analysis is prone to induce type I error. Therefore, trial sequential analysis (TSA) has been developed to reduce type I error (13). Meanwhile, TSA can determine whether a significant difference in the meta-analysis is conclusive (14).

Several previous systematic reviews (SRs) (15–19) already explored the effect of CIMT on patients with PSMD, but the conclusions were inconsistent. Due to differences in types of stroke, types of CIMT, and extremities, the previous SRs provided limited information for the clinical application of CIMT. In addition, the optimal protocol of CIMT for PSMD remains unclear. As more relevant clinical trials have been conducted in recent years, we performed this meta-analysis to comprehensively investigate the benefit of CIMT for PSMD and conducted TSA to assess the reliability of the main findings.

## 2. Methods

### 2.1. Study registration

The protocol of this meta-analysis and TSA has been registered on the International Prospective Register of Systematic Reviews (Registration ID: CRD42019143490). We conducted this meta-analysis and TSA strictly following A Measurement Tool to Assess Systematic Reviews (AMSTAR 2.0) (20) and reported the findings in accordance with the Preferred Reporting Items for Systematic Reviews and Meta-Analysis 2020 (PRISMA 2020) statement (21). The completed PRISMA checklist is shown in Supplementary material 1.

### 2.2. Literature and search strategy

A literature search was performed by two reviewers in the following databases: PubMed, Embase, the Cochrane Library, and Web of Science, from their inception to 1 January 2023. Search strategies were developed and tailored to the above databases by two professional librarians. Logical operators were used to combine Medical Subject Headings and free text words. The detailed search strategies for all databases are shown in Supplementary material 1. To identify possible eligible studies, we manually reviewed the gray literature and the reference lists of relevant studies. The websites of clinical trial registration were also searched.

### 2.3. Inclusion criteria

#### 2.3.1. Types of studies

We included RCTs that evaluated the effectiveness or/and safety of CIMT for PSMD. The published language was limited to English.

#### 2.3.2. Types of participants

Patients suffering from PSMD were included. The characteristics of eligible patients were as follows: (1) stroke was diagnosed according to the internationally accepted diagnostic criteria (22) or confirmed by magnetic resonance imaging (MRI)/computed tomography (CT) and (2) motor dysfunction was determined based on the Brunnstrom stage (stages III–IV) or validated scales. There were no restrictions on age, gender, race, and characteristics of stroke.

#### 2.3.3. Types of interventions

We included RCTs that used CIMT (e.g., CIMT and mCIMT) or CIMT combined with conventional rehabilitation (CR) as the experimental groups. There were no constraints on duration, frequency, and types of CIMT.

#### 2.3.4. Types of comparisons

Patients in the control group received CR (e.g., occupational therapy, physiotherapy, activity daily living training, and functional training) or usual care (UC).

#### 2.3.5. Types of outcome measures

The primary outcomes were a motor activity log for the amount of use (MAL-AOU) and quality of movement (MAL-QOM) scores. The secondary outcomes included the Fugl-Meyer assessment of upper extremity (FMA-UE), functional ability of the Wolf Motor Function Test (WMFT-FA), the Action Research Arm Test (ARAT) function, the Nine-Hole Peg Test (9HPT), gait parameters [e.g., step velocity (SV), step length (SL), and step width (SW)], hand function of the stroke impact scale (SIS-HF), and CIMT-related adverse events.

### 2.4. Exclusion criteria

Studies were excluded if they met any of the following conditions: (1) cross-over RCTs, quasi-RCTs, cohort studies, and case-control studies; (2) involved patients who suffered from motor dysfunction due to other diseases (e.g., cerebral palsy, traumatic brain injury, and Parkinson's disease); (3) unavailability of full text and data through various approaches; and (4) overlapping publications.

### 2.5. Study selection

All retrieved studies were imported into EndNote (X9). After removing duplicates, two independent reviewers screened the titles and abstracts to exclude irrelevant records. Then, the rest of the records with full text were further scrutinized to identify eligible studies. All the included studies were cross-checked. In the event of disagreement, a third author was consulted.

### 2.6. Data extraction

From the included studies, two reviewers extracted data independently using a predefined extraction form. The following information was extracted: (1) the information of studies; (2) the characteristics of participants; (3) the details of interventions; (4) the features of comparisons; and (5) outcome measures. After extraction, the two reviewers cross-checked the data to ensure accuracy. The Web Plot Digitizer was used to extract numerical data from figures. If the outcome-of-interest data were provided as means with 95% confidence intervals (CIs), standard errors (SE), or medians and interquartile ranges, we converted them into means and standard deviations (SD) according to the formula of the Cochrane Handbook or Wan et al.'s report, respectively (23, 24). We also contacted the corresponding authors for missing data. For multi-arm RCTs, we extracted the comparison with an inferior effect size to obtain more conservative results. We resolved disagreements through team discussion.

### 2.7. Assessment of reporting quality

Two independent reviewers utilized the Consolidated Standards of Reporting Trials 2010 (CONSORT 2010) to appraise the reporting quality of RCTs. The CONSORT 2010 consists of 37 items involving title and abstract (two items), introduction (two items), methods (17 items), results (10 items), discussion (three items), and other information (three items). Each item is assessed as either “yes” or “no”. Discrepancies were resolved by the third reviewer.

### 2.8. Risk-of-bias assessment

The revised Cochrane Collaboration risk-of-bias assessment tool for individually randomized, parallel-group trials, version 2.0 (ROB 2) (25) was used to evaluate the risk of bias in included studies. ROB 2 contains five domains; each domain is appraised as “low risk of bias,” “some concerns,” or “high risk of bias”. Two independent reviewers independently assessed the risk of bias and cross-checked, and any disagreement was arbitrated by a third reviewer.

### 2.9. Statistical analysis

The intraclass correlation coefficient (ICC) was calculated to evaluate the consistency between reviewers using the Statistical Package for Social Sciences (SPSS) 25.0. Consistency was defined as poor, fair to good, or excellent (26). Data synthesis was performed using RevMan software (version 5.4) and STATA software (version 13.0). The post-intervention data were synthesized. Since all the outcomes were continuous data, the results were presented as the weighted mean difference (MD) if the unit of measurement was consistent across studies; otherwise, we used the standardized mean difference (SMD). Heterogeneity among the studies was calculated using the chi-squared test and the *I*^2^ statistic. We used a random-effect model to pool data (27).

### 2.10. Subgroup analysis

We conducted a subgroup analysis for the primary outcome based on the following factors: the stroke stage, the restricted time of CIMT daily, the duration of CIMT, the types of CIMT, and follow-up time.

### 2.11. Sensitivity analysis

Sensitivity analysis was performed for the primary outcome by sequentially excluding studies one by one. In addition, we also conducted the sensitivity analysis after excluding studies with a high risk of bias.

### 2.12. Publication bias

The funnel plot, Begg's test, and Egger's test were used to detect the publication bias for the primary outcome when there were over 10 studies with the same outcome included in the analysis.

### 2.13. Trial sequential analysis

We applied TSA for primary outcome limiting to studies with a low risk of bias and some concerns using the TSA software (version 0.9.5.10-Beta). A random-effect model with a maximum type I error of 5% and a maximum type II error of 20% (80% power) was chosen. The cumulative z-score, monitoring boundary, futility boundary, and required information size (RIS) were presented in the TSA graph. If the included sample size reached the required information size or the cumulative Z curve crossed the monitoring boundary and futility boundary, the result was reliable.

### 2.14. Certainty of evidence

Two independent reviewers assessed the certainty of evidence using the Grading of Recommendations, Assessment, Development, and Evaluation (GRADE) system. The certainty of the evidence was graded “high,” “moderate,” “low,” or “very low”. The results of GRADE were cross-checked, and disagreements were resolved by team discussion. The GRADEpro (version 3.6) software was used to present the summary of the findings.

## 3. Results

### 3.1. ICC results

The results of ICC indicated excellent consistency in the processes of screening, extraction, and assessment of reporting quality, risk of bias, and certainty of evidence.

### 3.2. Search results

**Figure 1:**
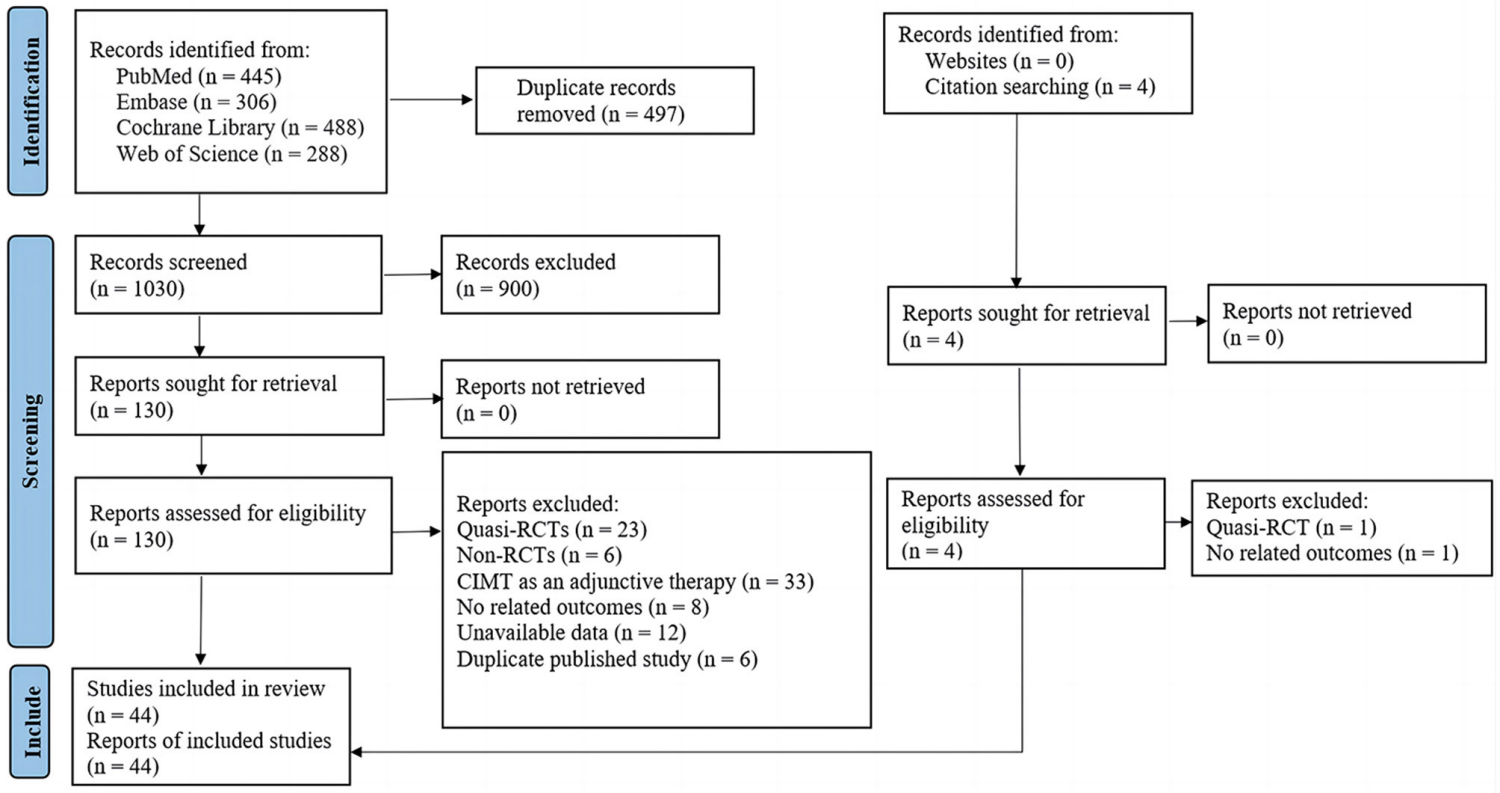
PRISMA flowchart.

As presented in Figure 1, we identified 1,527 records from electronic databases and citation searching. After removing duplicates and irrelevant records, we screened the remaining 130 studies in full text. A total of 44 eligible studies were included; the lists of included studies and those that were excluded with reasons are shown in Supplementary material 2.

### 3.3. Study characteristics

The included studies were published between 2000 and 2022. A total of 2,083 patients were involved, with ages ranging from 20 to 88. Patients in 24 studies suffered from stroke for the first time, and patients in three studies suffered from recurrent stroke; the remaining studies did not specify the number of strokes. Patients in six studies suffered from ischemic stroke, and the remaining studies included patients with both ischemic stroke and hemorrhagic stroke. In addition, patients in 11 studies suffered from a unilateral stroke, and the remaining studies did not report the exact location of the stroke. The duration of stroke varied from 1 day to 10 years; 25 studies included patients with acute and subacute stroke, and 19 studies included patients with chronic stroke. The basic characteristics of the included studies are shown in Supplementary Table 2. 25 studies used mCIMT and 19 studies used CIMT. The restriction time of CIMT varied from half an hour of restriction per day to 90% of the waking hours of restriction per day. The duration of CIMT ranged from 10 days to 60 days. The information in CIMT is summarized in Supplementary Table 3.

### 3.4. Assessment of reporting quality

The overall reporting percentage of included studies varied from 37.8 to 75.7%. According to CONSORT 2010, 14 of the 37 items (1b, 2a, 2b, 4a, 5, 6a, 8b, 12a, 13a, 14a, 16, 17a, 21, and 22) were adequately reported in all included studies, and four items (4b, 11a, 15, and 20) had a reporting rate over 70%. However, several items were inadequately reported, including 1a (52.2%, 23/44), 3a (50%, 22/44), 7a (34.1%, 15/44), 8a (15.9%, 7/44), 9 (31.8%, 14/44), 12b and 18 (4.5%, 2/44), 13b (47.7%, 21/44), 19 (11.4%, 5/44), 23 and 24 (18.2%, 8/44), and 25 (36.4%, 16/44). In addition, six items (3b, 6a, 7b, 10, 14b, and 17b) were not mentioned at all. The reporting quality of the CONSORT checklist is presented in Supplementary Tables 4, 5.

### 3.5. Risk-of-bias assessment

**Figure 2:**
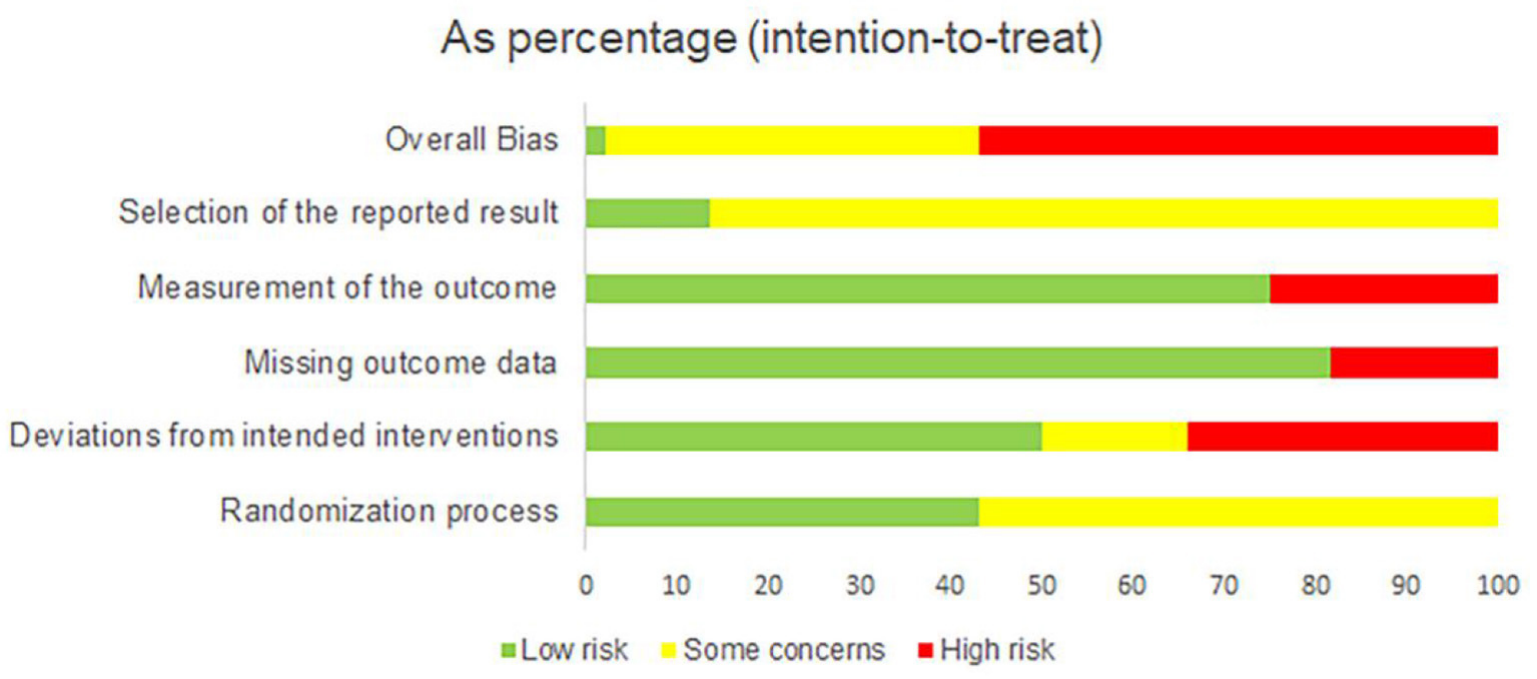
Risk-of-bias summary.

The summary of the ROB 2 assessment is shown in Figure 2, and a graph of the risk of bias is provided in Supplementary Figure 1. In the randomization process, 25 studies were evaluated as having some concerns as they did not provide information about allocation concealment. Regarding the deviation from intended interventions, 15 studies were evaluated as having a high risk of bias due to deviation from the intended intervention caused by the experimental context, no double-blinding method, and lack of intention-to-treat (ITT) analysis; seven studies did not use the double-blinding method and ITT analysis, which were evaluated as having some concerns. Regarding the missing outcome, eight studies did not provide the details of participant dropouts, which were evaluated as having a high risk of bias. Considering the measurement of outcomes, 11 studies were assessed as having a high risk of bias due to no blinding method of outcome assessors. For the selection of the reported result, 38 studies did not provide the protocol information and were thus evaluated as having some concerns. Overall, 25 studies were rated as having a high risk of bias, 18 studies were evaluated as having some concerns, and one was low.

### 3.6. Primary outcomes

#### 3.6.1. CIMT combined with CR vs. CR

**Figure 3:**
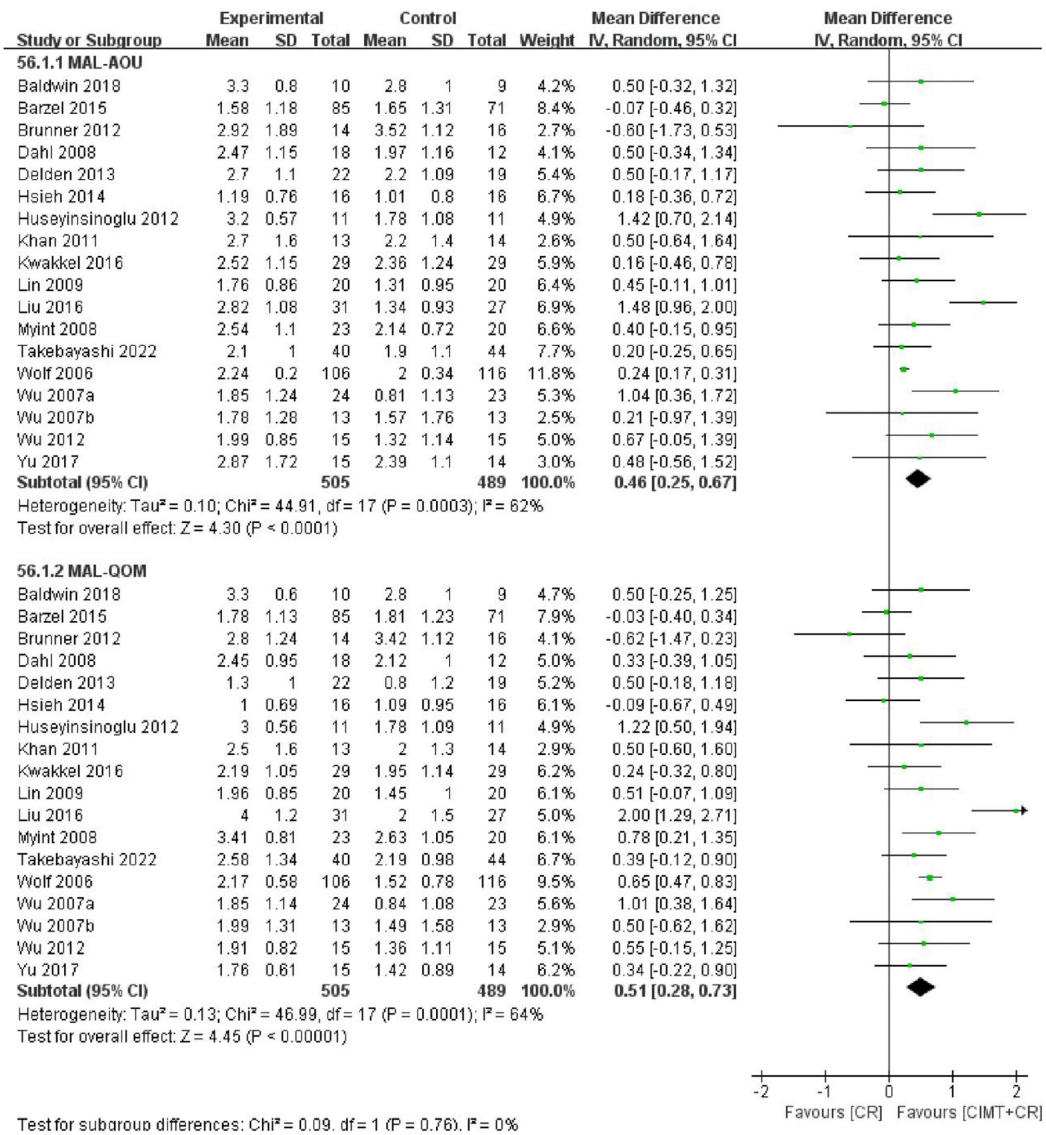
The forest plot of MAL comparing CIMT plus CR and CR.

**Figure 4:**
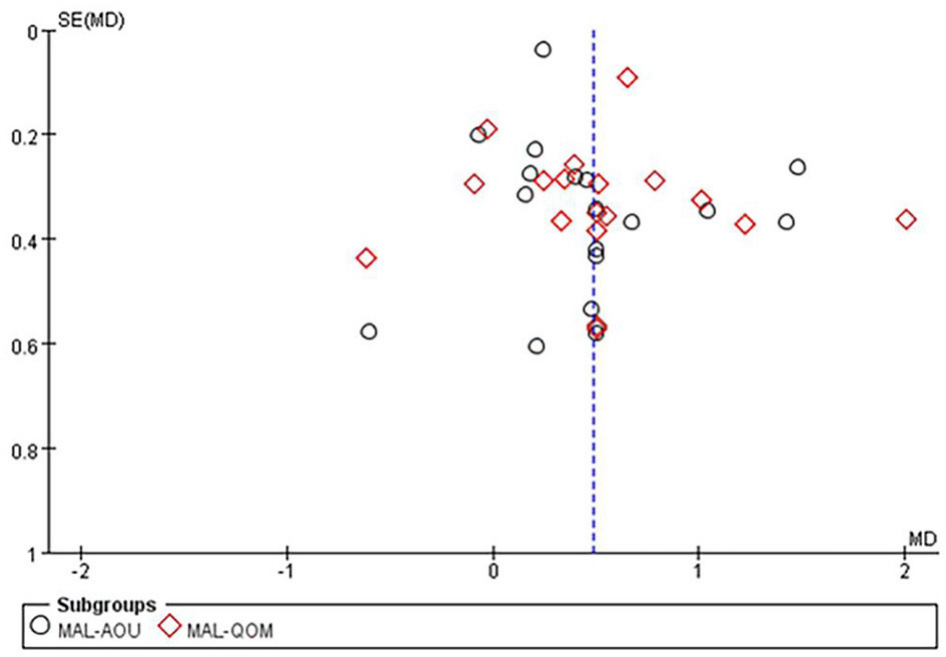
The funnel plot of MAL comparing CIMT plus CR and CR.

The MAL score was reported in 18 studies with 994 patients. The results of the meta-analysis showed that CIMT combined with CR had a greater effect than CR in improving motor function in patients after stroke (MAL-AOU: MD = 0.46, 95%CI = 0.25–0.67, *P* < 0.0001, *I*^2^ = 62%; MAL-QOM: MD = 0.51, 95%CI = 0.28–0.73, *P* < 0.00001, *I*^2^ = 64%) (Figure 3). The funnel plot, Begg's test (MAL-AOU: *P* = 0.503, MAL-QOM: *P* = 0.710), and Egger's test (MAL-AOU: *P* =0.720, MAL-QOM: *P* = 0.940) indicated that no obvious publication bias existed (Figure 4).

#### 3.6.2. CIMT vs. CR

**Figure 5:**
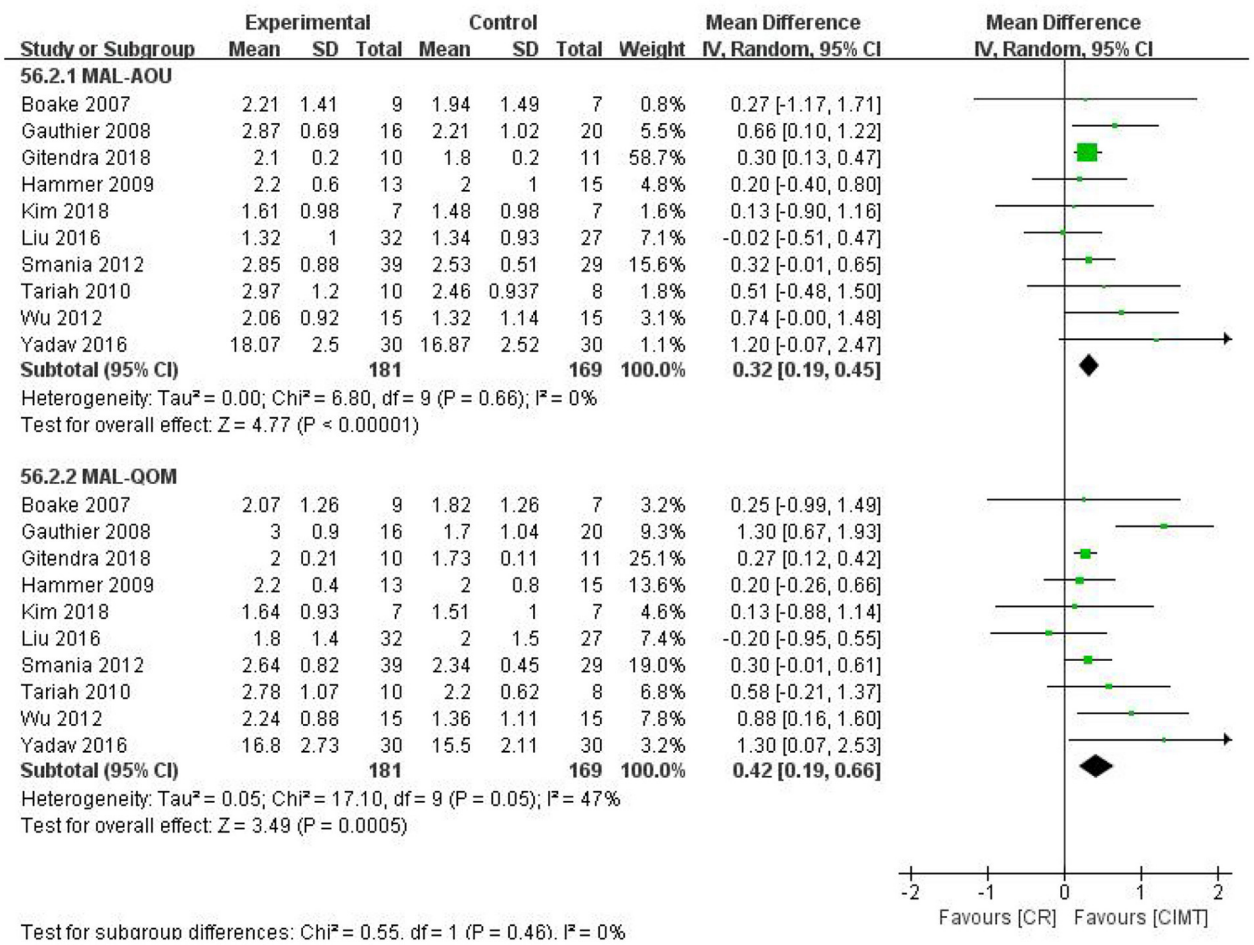
The forest plot of MAL comparing CIMT and CR.

**Figure 6:**
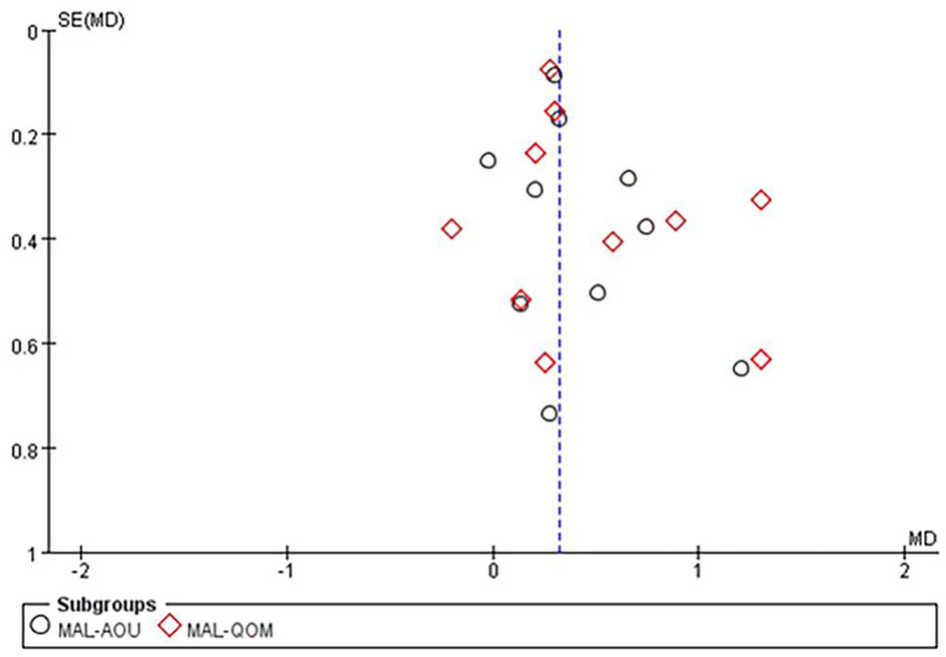
The funnel plot of MAL comparing CIMT and CR.

There were 10 studies that compared the effects of CIMT with CR. The pooled results revealed that CIMT was superior to CR in increasing the MAL score (MAL-AOU: MD = 0.32, 95%CI = 0.19–0.45, *P* < 0.00001, *I*^2^ = 0%; MAL-QOM: MD = 0.42, 95%CI = 0.19–0.66, *P* = 0.0005, *I*^2^ = 47%) (Figure 5). The funnel plot, Begg's test (MAL-AOU: *P* = 1, MAL-QOM: *P* = 1), and Egger's test (MAL-AOU: *P* = 0.380, MAL-QOM: *P* = 0.281) demonstrated no publication bias was detected (Figure 6).

#### 3.6.3. CIMT combined with CR vs. UC

**Figure 7:**
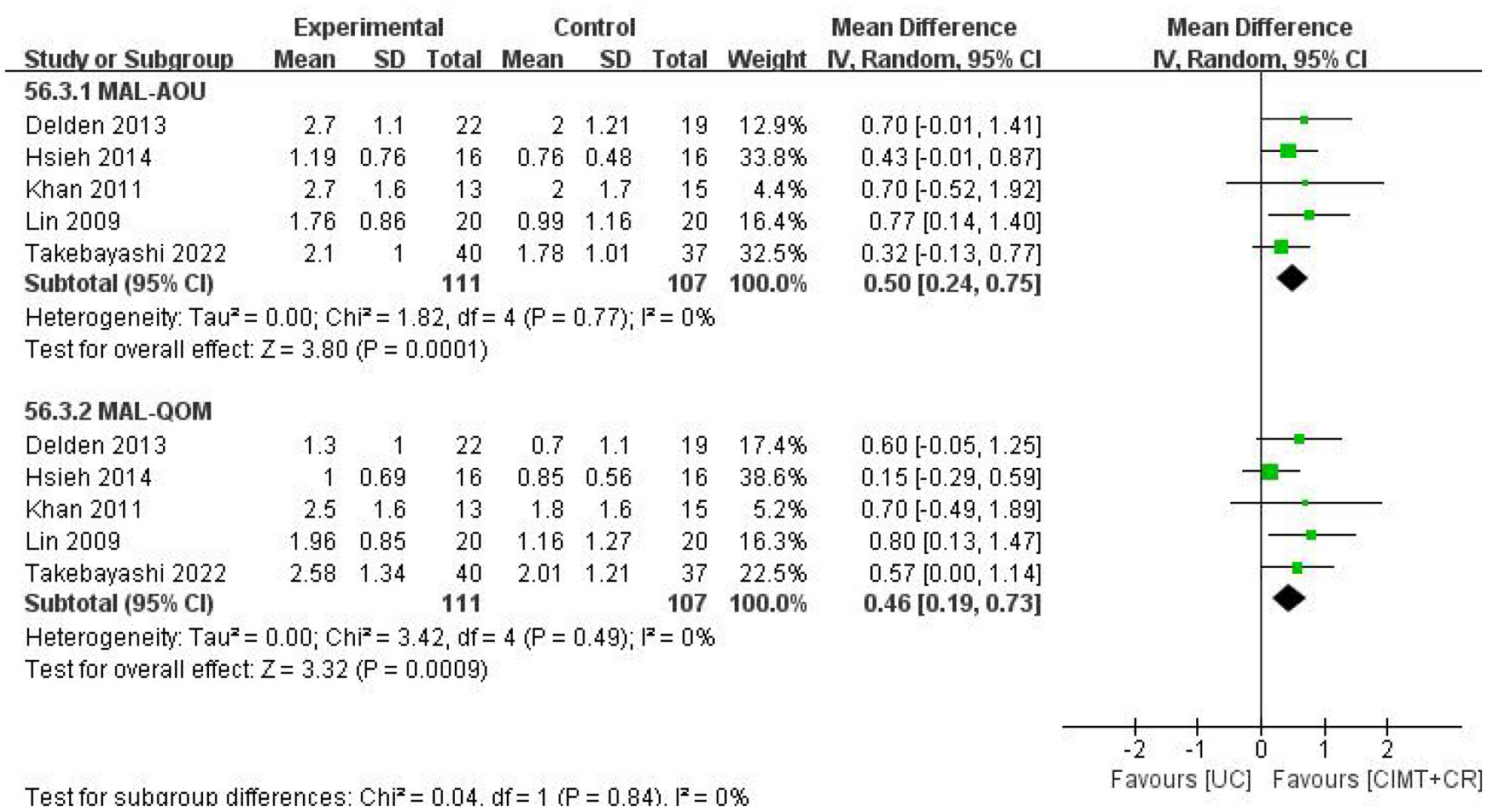
The forest plot of MAL comparing CIMT plus CR and UC.

There were five studies that compared CIMT plus CR vs. UC. The results manifested that CIMT combined with CR had a better effect than UC (MAL-AOU: MD = 0.50, 95%CI = 0.24–0.75, *P* = 0.0001, *I*^2^ = 0; MAL-QOM: MD = 0.46, 95%CI = 0.19–0.73, *P* =0.0009, *I*^2^ = 0) (Figure 7).

### 3.7. Subgroup analysis

The results of the subgroup analysis are summarized in Supplementary Table 6. In terms of the restricted time of CIMT daily, we found that ≥6 h of CIMT plus CR or CIMT alone were superior to CR in improving MAL scores. Regarding the total duration of CIMT, CIMT plus CR or CIMT ≤20 days were better than CR in enhancing MAL scores. For patients with stroke at any stage, CIMT combined with CR or CIMT had a better therapeutic effect than CR in improving MAL scores. Compared with CR, both mCIMT combined with CR or mCIMT and CIMT plus CR or CIMT increased greater MAL scores. The results of the subgroup analysis based on follow-up time showed that CIMT plus CR had a better effect ameliorating MAL scores after a stroke at a 1–3 month follow-up.

### 3.8. Sensitivity analysis

In terms of CIMT vs. CR, the synthesized results were reliable in the comparisons of CIMT combined with CR vs. CR, CIMT vs. CR, and CIMT plus CR vs. UC. The graphs of sensitivity analysis are provided in Supplementary Figures 2–7.

After excluding high-risk studies, the pooled results were also stable in CIMT combined with CR vs. CR, CIMT vs. CR, and CIMT plus CR vs. UC. The results are presented in Supplementary Figures 8–13.

### 3.9. Secondary outcomes

The results of secondary outcomes in the comparison of CIMT combined with CR vs. CR, CIMT vs. CR, and CIMT combined with CR vs. UC are shown in Supplementary Table 7.

#### 3.9.1. CIMT combined with CR vs. CR

##### 3.9.1.1. Fugl-Meyer assessment

There were 14 studies that observed the outcome of the FMA (FMA-UE) scores in the upper extremity. The results showed that CIMT combined with CR achieved better benefits than CR alone (MD = 2.42, 95%CI = 1.05–3.79, *P* = 0.0005, *I*^2^ = 0).

##### 3.9.1.2. Wolf motor function test

Compared with the CR group, participants in CIMT combined with the CR group had higher WMFT (WMFT-FA) scores in 14 studies (MD = 0.32, 95%CI = 0.12–0.51, *P* = 0.001, *I*^2^ = 50%).

##### 3.9.1.3. Action research arm test

There were eight studies with 310 patients that investigated the effect of CIMT combined with CR on the ARAT scores. The results of the meta-analysis showed that CIMT combined with CR had a better effect than CR (MD = 6.41, 95%CI = 4.48–8.33, *P* < 0.00001, *I*^2^ = 0).

##### 3.9.1.4. FIM

In terms of the FIM scores, there were no significant differences between CIMT combined with the CR group and the CR group in 7 studies (SMD = 0.66, 95%CI = −0.16–1.48, *P* = 0.11, *I*^2^ = 87%).

##### 3.9.1.5. Nine-hole peg test (9HPT)

The pooled data from six studies revealed that CIMT combined with CR was not better than CR in the 9HPT scores (MD = −0.01, 95%CI = −0.05–0.02, *P* = 0.53, *I*^2^ = 2%).

##### 3.9.1.6. Hand function of stroke impact scale (SIS-HF)

The SIS-HF was reported in eight trials, and the synthesized results showed there were no significant differences between CIMT combined with the CR group and the CR group in SIS-HF scores (MD = 0.49, 95%CI = −1.78–2.76, *P* = 0.67, *I*^2^ = 8%).

##### 3.9.1.7. Gait parameters

CIMT combined with the CR group was superior to the CR group in improving gait parameters (SV: MD = 0.10, 95%CI = 0.01–0.18, *P* = 0.03, *I*^2^ = 0; SL: MD = 0.09, 95%CI = 0.03–0.14, *P* = 0.001, *I*^2^ = 0; SW: MD = 0.03, 95%CI = 0.01–0.05, *P* = 0.002, *I*^2^ = 0).

#### 3.9.2. CIMT vs. CR

##### 3.9.2.1. Fugl-Meyer assessment of upper extremity (FMA-UE)

The results of a meta-analysis of 11 studies showed that CIMT had a better effect on increasing the FMA-UE scores of the upper extremity than CR (MD = 2.98, 95%CI = 1.58–4.37, *P* < 0.0001, *I*^2^ = 41%).

##### 3.9.2.2. Functional ability of wolf motor function test (WMFT-FA)

The pooled data from six studies demonstrated that there was a significant difference between the CIMT group and the CR group in enhancing WMFT-FA scores (MD = 0.42, 95%CI = 0.18–0.65, *P* = 0.0005, *I*^2^ = 0).

##### 3.9.2.3. Action research arm test

The ARAT scores were reported in four studies with 211 patients. The pooled results showed that the CIMT group was more effective than the CR group (MD = 9.82, 95%CI = 3.70–15.94, *P* = 0.002, *I*^2^ = 81%).

##### 3.9.2.4. FIM

In terms of FIM scores, the results of the meta-analysis showed that CIMT was not superior to CR (SMD = 1.07, 95%CI = −1.53–3.67, *P* = 0.42, *I*^2^ = 93%).

#### 3.9.3. CIMT combined with CR vs. UC

##### 3.9.3.1. Fugl-meyer assessment of upper extremity (FMA-UE)

The synthesized results from six studies revealed that CIMT combined with CR achieved better benefits than UC in increasing the FMA (FMA-UE) scores (MD = 3.64, 95%CI = 1.87–5.41, *P* < 0.0001, *I*^2^ = 3%).

##### 3.9.3.2. Functional ability of wolf motor function test

Compared with the UC group, participants in CIMT combined with the CR group had greater WMFT (WMFT-FA) scores (MD = 0.28, 95%CI = 0.01–0.58, *P* = 0.02, *I*^2^ = 17%).

##### 3.9.3.3. Action research arm test

The results of the meta-analysis showed that CIMT combined with the CR group had a better effect than the UC group in increasing the ARAT scores (MD = 8.12, 95%CI = 5.70–10.55, *P* < 0.00001, *I*^2^ = 21%).

##### 3.9.3.4. FIM

There was no significant difference between CIMT combined with the CR group and the UC group in enhancing the FIM scores (SMD = 0.83, 95%CI = −0.18–3.49, *P* = 0.06, *I*^2^ = 88%).

##### 3.9.3.5. Stroke impact scale

The pooled data from two trials discovered that CIMT combined with the CR group was not better than the UC group in improving the SIS (SIS-HF) scores (MD = 9.26, 95%CI = −0.94–19.46, *P* = 0.08, *I*^2^ = 0).

### 3.10. Adverse events

The reports of thirteen studies showed no CIMT-related adverse events. Wolf reported (28) that a recurrent stroke occurred in the CIMT plus CR group. Thrane (29) observed shoulder pain during the treatment in the CIMT combined with the CR group.

### 3.11. Trial sequential analysis

After excluding high-risk studies in terms of TSA, in the comparison of CIMT combined with CR vs. CR, CIMT vs. CR, and CIMT plus CR vs. UC, their cumulative Z curves crossed the monitoring boundary and futility boundary. Meanwhile, the included sample sizes reached the RIS when comparing CIMT combined with CR and CR. Therefore, the results indicated that there was sufficient evidence to support the effects of CIMT combined with CR and CIMT alone in PSMD. The graphs of TSA are provided in Supplementary Figures 14–19.

### 3.12. Certainty of evidence

As shown in Supplementary Figures 20–22, the score of 9HPT and gait parameters in comparison of CIMT combined with CR vs. CR were evaluated as having a “moderate” certainty of the evidence, while the remaining outcomes were considered as “low” or “very low”. The main downgrading factors were the high risk of bias, imprecision, inconsistency, and publication bias in the included studies.

## 4. Discussion

### 4.1. The effect of CIMT on PSMD

In this meta-analysis and TSA, we found that CIMT alone or CIMT combined with CR could effectively increase MAL, FMA, WMFT, and ARAT scores and improve gait parameters. The above evidence was reliable according to TSA. The results demonstrated that CIMT could improve PSMD, which was consistent with the previous SRs. Meanwhile, Lang et al. (30) reported that the minimum clinically important difference (MCID) of MAL-AOU and MAL-QOM with moderate clinical significance and high clinical significance were 0.2/0.2 and 0.5/0.5, respectively. According to our results, the MDs of MAL-AOU and MAL-QOM (CIMT alone, CIMT combined with CR) were 0.32/0.46 and 0.32/0.51, respectively, which indicated that the effect of CIMT alone or combined with CR was clinically effective in PSMD. In addition, CIMT as a neuromotor therapy for PSMD could increase dendritic spine density and the plasticity in the contralateral sensorimotor cortex (31). Page et al. (32) used focal transcranial magnetic stimulation to monitor the change in the cortical motor area in patients with PSMD before and after CIMT and found that the repetitive use of affected limbs could enhance cortical reorganization and then improve PSMD. It was reported that brain-derived neurotrophic factors increased in patients with stroke after CIMT treatment, which could promote the development and recovery of brain motor neurons and improve PSMD (33). Tang et al. (34) found that CIMT increased the expression of glutamate receptors (GluR1, GluR2, and NR1) in patients with stroke. Moreover, CIMT can increase the complexity and density of dendritic in the residual cortex and hippocampus in patients with PSMD (35). Therefore, we speculated that CIMT overcame a learned non-use phenomenon by increasing the use of the affected upper limbs and enhancing the motor automaticity of the affected limbs during treatment. However, the underlying neuroplastic mechanism of CIMT for PSMD warrants further investigation.

### 4.2. Compared with previous reviews

Liu et al. (16) included 16 identified RCTs that investigated the effect of CIMT alone or mCIMT alone on motor dysfunction in patients with acute and subacute stroke and concluded that CIMT or mCIMT might be more beneficial than CR. Meanwhile, McIntyre et al. (17) found that CIMT or mCIMT was superior to CR in improving motor function of the upper extremity for patients with chronic stroke. Based on 13 RCTs, Shi et al. (18) concluded that mCIMT could improve the ability to use the paretic upper extremity. Tedla et al. (19) focused on the effect of CIMT or mCIMT on motor dysfunction of the lower extremity and revealed a positive effect of CIMT or mCIMT on balance function but not on functional mobility. Etoom et al. (15) included 36 RCTs to investigate the effect of CIMT or mCIMT on patients with stroke who had upper limb dysfunction, and the results showed that there was weak evidence for the superiority of CIMT/mCIMT in comparison with CR in PSMD. In the present study, we included 44 RCTs and comprehensively evaluated the effect of CIMT or mCIMT on PSMD. To identify optimal protocol, we conducted a subgroup analysis according to the restricted time of CIMT daily, the total restricted days of CIMT, the stroke stage, and the types of CIMT. Moreover, TSA was performed to assess the reliability of the main findings.

### 4.3. The influence factors on the effect of CIMT

#### 4.3.1. Different CIMT types

Our results indicated that CIMT alone or CIMT plus CR and mCIMT alone or mCIMT plus CR had more benefits than CR in alleviating PSMD. Nevertheless, an increasing number of studies recommend mCIMT instead of CIMT as an intervention for patients with PSMD. CIMT requires long daily restricted time (90% of daily waking hours), which is associated with poor treatment compliance (36). In contrast, mCIMT shortens the restraint time of the unaffected limbs (6 h per day) and requires less intensive training sessions for the affected limbs, which is more acceptable for patients with stroke (37). Furthermore, the results of the subgroup analysis showed that mCIMT alone or mCIMT plus CR had a higher effect size on MAL scores; thus, we preferred to recommend mCIMT for patients with PSMD.

#### 4.3.2. Different daily restricted time and total restricted days

Sirtori et al. (38) discovered that the prescribed treatment time of CIMT affected the recovery of PSMD. The results of subgroup analysis demonstrated that CIMT of ≥6 h was better than CR in alleviating PSMD. While CIMT of 1–5 h per day had no significant difference, the reason may be related to the time-superimposed effects of CIMT (39). Moreover, our results suggested that the duration of CIMT of more than 20 days was ineffective for improving PSMD. One possible explanation was that the long duration of CIMT may lead to poor patient compliance and an unsustainable therapeutic effect (40). A review concluded that a significant improvement in PSMD was observed in CIMT with a duration of fewer than 14 days (30 h) but not in CIMT with a duration of over 14 days (30 h) (38). Nonetheless, due to high heterogeneity and high risk of bias, the above results need further verification.

#### 4.3.3. Different stroke stages

We found that CIMT alone or CIMT combined with CR had a better therapeutic effect than CR to ameliorate motor function at the acute, subacute, or chronic stages of stroke. Preclinical studies (41, 42) revealed that there might be a time-limited window of brain neuroplasticity following the acute and subacute stages of stroke, during which rehabilitation is most effective. However, several studies (43, 44) concluded that CIMT was not suitable for patients with stroke in the acute and subacute stages because CIMT that started in the early days after stroke might aggravate PSMD. More studies are required to address this issue.

Except for the abovementioned influencing factors, sleep quality and stroke location might also have an impact on the effect of CIMT. Pereira et al. (45) investigated the influence of sleep quality on the effect of CIMT for PSMD, and the results showed that patients with poor sleep quality had less improvement in motor performance than those with good sleep quality. Vidal et al. (46) found that, compared with patients with left hemispheric stroke, those with right hemispheric stroke had better sensorimotor cortex activation after CIMT. Due to limited studies, the optimal protocol and influencing factors of CIMT remain to be investigated.

### 4.4. Strengths and limitations

This is the latest meta-analysis and TSA of CIMT for PSMD, and we performed TSA to control for random error and explore the reliability of the main finding (13). We included RCTs aiming to achieve reliable evidence. The protocol for this meta-analysis and TSA was registered in advance. This meta-analysis and TSA were conducted strictly according to AMSTAR 2.0 and reported in accordance with PRISMA 2020. In addition, we used ROB2 and CONSORT 2010 to comprehensively assess the risk of bias and reporting quality of included studies. Nevertheless, there are also several limitations. First, as the published language of the RCTs included in this meta-analysis and TSA was limited to English, language bias was inevitable. Second, due to high or some concerns of risk of bias, the results should be treated with caution. Third, we used MAL as the primary outcome, which is a subjective assessment scale, and although we pooled data from studies with blinding outcome assessors, measurement bias might still exist.

## 5. Conclusion

Constraint-induced movement therapy may be an optional and safe rehabilitation therapy to improve PSMD. However, due to limited studies, the optimal protocol of CIMT for PSMD was not determined, and more RCTs are required for further exploration.

## Data availability statement

The original contributions presented in the study are included in the article/Supplementary material, further inquiries can be directed to the corresponding authors.

## Author contributions

JZ, XX, and QJ contributed equally to this study, wrote, and edited the manuscript. RJ and JL conceptualized the study and provided methodological support. XL and CX designed the search strategy and extracted the data. YL and DZ selected the studies. QJ and XX assessed the risk of bias. JZ and YQ rated the certainty of evidence. XL, CX, and YL analyzed the data. All authors approved the manuscript.
