# Supplementary material for: The effect and safety of constraint-induced movement therapy for post-stroke motor dysfunction: a meta-analysis and trial sequential analysis
Source: Front Neurol. 2023 Apr 18;14:1137320. doi: 10.3389/fneur.2023.1137320 (PMC10151521; doi:10.3389/fneur.2023.1137320)
Supplement: Supplementary file 2 [file Data_Sheet_2.ZIP › Table S1-S7.docx]

**Table S1: The list of excluded records**

| No. | First Author | Title | Excluded Reason |
| --- | --- | --- | --- |
| 1 | Abbasi, S | The effects of Constraint-Induced therapy on strength and dexterity of upper extremity in adult hemiplegic patients | Quasi-RCTs |
| 2 | Angerova, Y | Treatment programme for lower limb central paresis based on constrained induced movement therapy | Quasi-RCTs |
| 3 | Angerova, Y | Long-term results of constraint induced movement therapy in day programme for people with acquired brain injury | Quasi-RCTs |
| 4 | Angerova, Y | Long lasting effect of day program with CIMT (Constraint Induced Movement Therapy) in stroke patiens | Quasi-RCTs |
| 5 | Bonifer, N. M | Constraint-induced movement therapy after stroke: Efficacy for patients with minimal upper-extremity motor ability | Quasi-RCTs |
| 6 | Bonnyaud, C | Effects of a constraint-induced therapy on gait biomechanics parameters in hemiparetic patients after overground or treadmill training | Quasi-RCTs |
| 7 | Corbetta, D | Constraint-induced movement therapy for upper extremities in people with stroke | Quasi-RCTs |
| 8 | Dettmers, C | Distributed form of constraint-induced movement therapy improves functional outcome and quality of life after stroke | Quasi-RCTs |
| 9 | Doussoulin, A | Recovering functional independence after a stroke through Modified Constraint-Induced Therapy | Quasi-RCTs |
| 10 | Galvao, F. R. O | Group-based constraint-induced movement therapy in the rehabilitation of chronic poststroke patients | Quasi-RCTs |
| 11 | Gauthier, L | Delivery of constraint-induced movement therapy through a video game: A pilot study in stroke | Quasi-RCTs |
| 12 | Hosomi, M | A modified method for constraint-induced movement therapy: A supervised self-training protocol | Quasi-RCTs |
| 13 | Hu, J | Constraint-induced movement therapy improves functional recovery after ischemic stroke and its impacts on synaptic plasticity in sensorimotor cortex and hippocampus | Quasi-RCTs |
| 14 | Jaffe, J | Delivery of constraint-induced movement therapy through a video game for individuals with hemiparesis post-stroke | Quasi-RCTs |
| 15 | Kallio, K | Modified constraint-induced therapy for the lower extremity in elderly persons with chronic stroke: Single-subject experimental design study | Quasi-RCTs |
| 16 | Matuti, G | Immediate and Long-term Effects of Constraint Induced Movement Therapy in Traumatic Brain Injury Patients | Quasi-RCTs |
| 17 | McCall, M | Modified constraint-induced movement therapy for elderly clients with subacute stroke | Quasi-RCTs |
| 18 | Miltner, W. H. R | Effects of constraint-induced movement therapy on patients with chronic motor deficits after stroke: A replication | Quasi-RCTs |
| 19 | Page, S. J | Modified Constraint-Induced Therapy Extension: Using Remote Technologies to Improve Function | Quasi-RCTs |
| 20 | Page, S. J | Modified constraint-induced therapy in patients with chronic stroke exhibiting minimal movement ability in the affected arm | Quasi-RCTs |
| 21 | Page, S. J | Modified constraint induced therapy: A randomized feasibility and efficacy study | Quasi-RCTs |
| 22 | Page, S. J | Modified constraint-induced therapy in chronic stroke | Quasi-RCTs |
| 23 | Parneix, E | Comparing pre-post-treatment effects of constraint induced movement therapy (CIMT) in a patient after stroke: A qualitative analysis of performance in instrumental activities of daily living using the situational assessment AMPS | Quasi-RCTs |
| 24 | Pierce, S. R | Home Forced Use in an Outpatient Rehabilitation Program for Adults with Hemiplegia: A Pilot Study | Quasi-RCTs |
| 25 | Tarkka, I. M | Alterations in cortical excitability in chronic stroke after constraint-induced movement therapy | Non-RCT |
| 26 | Tarkka, I. M | Paretic hand rehabilitation with constraint-induced movement therapy after stroke | Non-RCT |
| 27 | Taub, E | Constraint-induced movement therapy to enhance recovery after stroke | Non-RCT |
| 28 | Taub, E | Constraint induced movement techniques to facilitate upper extremity use in stroke patients | Non-RCT |
| 29 | Uswatte, G | Constraint-induced movement therapy for upper-extremity hemiparesis after stroke: ignoring degree of fidelity to the treatment protocol contributes to underestimate of effect size in meta-analyses | Non-RCT |
| 30 | Wang, W | Constraint-induced movement therapy promotes brain functional reorganization in stroke patients with hemiplegia | Non-RCT |
| 31 | Bang, D. H | The effects of modified constraint-induced movement therapy combined with trunk restraint in subacute stroke: a double-blinded randomized controlled trial | CIMT as an adjunctive therapy |
| 32 | Carrico, C | Randomized Trial of Peripheral Nerve Stimulation to Enhance Modified Constraint-Induced Therapy After Stroke | CIMT as an adjunctive therapy |
| 33 | Pernambuco  UFD | Effects of tDCS Combined With mCIMT or Mental Practice in Poststroke Patients | CIMT as an adjunctive therapy |
| 34 | Levy C E | Botulinum toxin a, evidence-based exercise therapy, and constraint-induced movement therapy for upper-limb hemiparesis attributable to stroke: a preliminary study | CIMT as an adjunctive therapy |
| 35 | Fan, L. B | Application of electroacupuncture plus movement therapy in recovering neurologic function of patients with spastic hemiplegia | CIMT as an adjunctive therapy |
| 36 | Figlewski, K | Transcranial direct current stimulation combined with constraint induced movement therapy facilitates motor function in chronic stroke patients | CIMT as an adjunctive therapy |
| 37 | Kim S H | The effect of modified Constraint-Induced Movement Therapy and dual Transcranial direct current stimulation on the recovery of upper limb function in chronic stroke patients | CIMT as an adjunctive therapy |
| 38 | Kim, H | The effects of mental practice combined with modified constraint-induced therapy on corticospinal excitability, movement quality, function, and activities of daily living in persons with stroke | CIMT as an adjunctive therapy |
| 39 | Lisa, S | Sitting Balance Exercise Performed Using Virtual Reality Training on a Stroke Rehabilitation Inpatient Service: a Randomized Controlled Study | CIMT as an adjunctive therapy |
| 40 | Nasb, M | Comparison of the effects of modified constraint-induced movement therapy and intensive conventional therapy with a botulinum-a toxin injection on upper limb motor function recovery in patients with stroke | CIMT as an adjunctive therapy |
| 41 | Page, S. J | Back from the brink: Electromyography-triggered stimulation combined with modified constraint-induced movement therapy in chronic stroke | CIMT as an adjunctive therapy |
| 42 | Rocha, S | The impact of transcranial direct current stimulation (tDCS) combined with modified constraint-induced movement therapy (mCIMT) on upper limb function in chronic stroke: a double-blind randomized controlled trial | CIMT as an adjunctive therapy |
| 43 | Shih, T. Y | A kinematic study of combined robot-aided arm therapy and constraint-induced therapy in chronic stroke | CIMT as an adjunctive therapy |
| 44 | Siqueira, A. O | Constraint-induced movement therapy and mental practice to improve arm function in stroke patients | CIMT as an adjunctive therapy |
| 45 | Song, Y | Combined rehabilitation with scalp cluster acupuncture and constraint-induced movement therapy significantly improved functional recovery in patients with acute ischemic stroke | CIMT as an adjunctive therapy |
| 46 | Sun, S. F | Combined botulinum toxin type A with modified constraint-induced movement therapy for chronic stroke patients with upper extremity spasticity: a randomized controlled study | CIMT as an adjunctive therapy |
| 47 | Yang, J. S | Scalp acupuncture combined with modified constraint exercise therapy for the rehabilitation of upper limb function in patients with stroke | CIMT as an adjunctive therapy |
| 48 | Abdullahi, A | Comparing Two Different Modes of Task Practice during Lower Limb Constraint-Induced Movement Therapy in People with Stroke: A Randomized Clinical Trial | CIMT as an adjunctive therapy |
| 49 | Brogardh, C | Constraint-induced movement therapy in patients with stroke: A pilot study on effects of small group training and of extended miit use | CIMT as an adjunctive therapy |
| 50 | Chen, H. C | Very early constraint-induced movement therapy (VECTORS): Effects of timing or dose on upper extremity function | CIMT as an adjunctive therapy |
| 51 | Eras-Garcia, R | Effects Of Modified Constraint-Induced Movement Therapy On Post-Stroke Functional And Occupational Performance | CIMT as an adjunctive therapy |
| 52 | Fuzaro, A. C | Modified constraint-induced movement therapy and modified forced-use therapy for stroke patients are both effective to promote balance and gait improvements | CIMT as an adjunctive therapy |
| 53 | Gauthier, L. V | Video Game Rehabilitation for Outpatient Stroke (VIGoROUS): Protocol for a multi-center comparative effectiveness trial of in-home gamified constraint-induced movement therapy for rehabilitation of chronic upper extremity hemiparesis | CIMT as an adjunctive therapy |
| 54 | Ju, Y | The effects of modified constraint-induced movement therapy and mirror therapy on upper extremity function and its influence on activities of daily living | CIMT as an adjunctive therapy |
| 55 | Lima, R | The short-and long-term benefits of home-based constraint induced movement therapy in individuals with chronic stroke | CIMT as an adjunctive therapy |
| 56 | Pactr | Constraints induced movement therapy for lower limb | CIMT as an adjunctive therapy |
| 57 | Rivas, R. J | Application of constraint induced movement therapy protocol: Effectiveness on the quality and quantity of upper extremity movement recovery after stroke | CIMT as an adjunctive therapy |
| 58 | Saruhan, Z | The effect of constraint-induced movement therapy for upper extremity on activities of daily living and neuronal plasticity in stroke patients | CIMT as an adjunctive therapy |
| 59 | Sirtori, V | Constraint-induced movement therapy for upper extremities in stroke patients | CIMT as an adjunctive therapy |
| 60 | Sterr, A | Longer versus shorter daily constraint-induced movement therapy of chronic hemiparesis: An exploratory study | CIMT as an adjunctive therapy |
| 61 | Stock, R | Early versus late-applied constraint-induced movement therapy: a multisite, randomized controlled trial with a 12-month follow-up | CIMT as an adjunctive therapy |
| 62 | Takebayashi, T | Efficacy of hybrid constraint-induced movement therapy | CIMT as an adjunctive therapy |
| 63 | Zhang, L. F | Effect of Scalp Electroacupuncture Combined Constraint-induced Movement Therapy on Move- ment Function of Ischemic Stroke Patients' Upper Limbs | CIMT as an adjunctive therapy |
| 64 | Anjos, S. D | Lower Extremity Constraint-Induced Movement Therapy (LE-CIMT) to Improve Gait and Mobility of People with Stroke | No related outcomes |
| 65 | Azab, M | Effectiveness of constraint-induced movement therapy (CIMT) as home-based therapy on Barthel Index in patients with chronic stroke | No related outcomes |
| 66 | Bhattacharjee, P | Effect of modified constraint induced movement therapy in improving upper extremity functions in hemiplegia | No related outcomes |
| 67 | Cavazza, S | Transcranial magnetic stimulation mapping and upper limb dexterity in subacute hemiplegic patients after treatment with Constraint Induced Movement Therapy | No related outcomes |
| 68 | Chi, Ctr Ior | Modified constraint-induced movement therapy in early stroke rehabilitation and brain plasticity | No related outcomes |
| 69 | Marandola, M. M | Constraint-induced movement therapy in the rehabilitation of hemineglect after a stroke | No related outcomes |
| 70 | Weng, C. S | Effects of constraint-induced movement therapy on upper limb function in sub-acute stroke patients | No related outcomes |
| 71 | Wittenberg, G. F | Constraint-induced therapy in stroke: Magnetic-stimulation motor maps and cerebral activation | No related outcomes |
| 72 | Yumi, J. U | The effects of modified constraint-induced movement therapy and mirror therapy on upper extremity function and its influence on activities of daily living | No related outcomes |
| 73 | Atteya, A. A. A | Effects of modified constraint induced therapy on upper limb function in subacute stroke patients | Unavailable data |
| 74 | Boe, S | Feasibility and effectiveness of modified constraint induced movement therapy in acute stroke rehabilitation | Unavailable data |
| 75 | Dincer, U | The effect of modified constraint induced therapy on upper limb function in patients with subacute and chronic stroke | Unavailable data |
| 76 | Amini, N | The effect of Constraint induced movement and traditional rehabilitation on chronic hemiplegic patient | Unavailable data |
| 77 | Li, H | Evaluation on curative effect of modified constraint-induced movement therapy in rehabilitation of activity of daily living in patients with sub-acute stroke | Unavailable data |
| 78 | Li, Z. L | Recovery effect of constraint-induced movement therapy on supper extremity activity of stroke patients with hemiparasis | Unavailable data |
| 79 | Pedlow, K | Constraint induced movement therapy (CIMT) for patients post acquired brain injury: Quantitative results from a clinical feasibility randomised controlled trial (RCT) | Unavailable data |
| 80 | Singh, P | Study to assess the effectiveness of modified constraint-induced movement therapy in stroke subjects: A randomized controlled trial | Unavailable data |
| 81 | Zhang, W. M | Effect of modified constraint-induced movement therapy on the activities of daily living of patients with acute stroke | Unavailable data |
| 82 | Page, S.J. | Modified Constraint-Induced Therapy after Subacute Stroke: A Preliminary Study | Unavailable data |
| 83 | Page, S.J. | Efficacy of Modified Constraint-Induced Movement Therapy in Chronic Stroke: A Single-Blinded Randomized Controlled Trial | Unavailable data |
| 84 | Page, S.J. | Modified Constraint-Induced Therapy in Acute Stroke: A Randomized Controlled Pilot Study | Unavailable data |
| 85 | Wu, C.Y. | Effects of modified constraint-induced movement therapy on movement kinematics and daily function in patients with stroke: a kinematic study of motor control mechanisms | Duplicate published study |
| 86 | Lin, K.C. | Effects of modified constraint-induced movement therapy on reach-to-grasp movements and functional performance after chronic stroke: a randomized controlled stud | Duplicate published study |
| 87 | Lin, K.C. | A randomized controlled trial of constraint-induced movement therapy after stroke | Duplicate published study |
| 88 | Lin, K.C. | Constraint-induced therapy versus dose-matched control intervention to improve motor ability, basic/extended daily functions, and quality of life in stroke | Duplicate published study |
| 89 | Lin, K.C. | Constraint-induced therapy versus control intervention in patients with stroke: a functional magnetic resonance imaging study | Duplicate published study |
| 90 | Wu, C.Y. | Randomized trial of distributed constraint-induced therapy versus bilateral arm training for the rehabilitation of upper-limb motor control and function after stroke | Duplicate published study |

**Table S2: Study Characteristics of Included Studies**

| Included studies | Country | Sample size  (E/C) | Female  (%) | Mean  age  (E/C) | Type of stroke  (E/C) | First  onset of stroke | Unilateral stroke | Mean  duration  of stroke  (E/C) | Experimental group | Control  group | Outcomes | Adverse effect | Follow-up |
| --- | --- | --- | --- | --- | --- | --- | --- | --- | --- | --- | --- | --- | --- |
| CIMT for upper extremity motor dysfunction after stroke | | | | | | | | | | | | | |
| Dromerick 2000 | America | 20(11/9) | 45 | E:61.5±13.7  C:71.4±5.3 | ischemia | / | / | 1-14d | CIMT+CR | CR | ④⑤ | / | / |
| Suputtitada2004 | Thailand | 69(33/36) | 32 | E:73.5±6.35  C:67.4±13.81 | ischemia  & hemorrhage | N | / | 1-10y | CIMT | CR | ⑤ | / | / |
| Ploughman2004 | Canada | 23(10/13) | 65 | E:57.8±10.65  C:61.62±5.86 | ischemia  & hemorrhage | Y | / | 1-16w | CIMT | CR | ④⑤ | N | / |
| Wolf  2006 | America | 222(106/116) | 35 | E:61±13.5  C:63.4±12.6 | ischemia  & hemorrhage | Y | / | 3-9m | CIMT+CR | CR | ①③⑦ | Y | Y |
| Wu  2007a | China | 47(24/23) | 32 | E:53.9±11.2  C:56.8±12.9 | ischemia  & hemorrhage | Y | / | 3w-37m | CIMT+CR | CR | ①② | / | / |
| Wu  2007b | China | 26(13/13) | 42 | E:71.44±6.42  C:71.94±16.7 | ischemia  & hemorrhage | Y | / | 0.5-31m | CIMT+CR | CR | ①②④⑦ | / | / |
| Boake 2007 | America | 16(9/7) | 50 | E:63.1±6.29 C:58.9±14 | ischemia  & hemorrhage | Y | / | 1-14d | CIMT | CR | ①② | N | Y |
| Gauthier 2008 | America | 36(16/20) | 47 | 63.3±12 | ischemia  & hemorrhage | / | / | 1-3.6y | CIMT | CR | ①③ | / | / |
| Myint 2008 | China | 43(23/20) | 56 | E:63.4±13.6 C:63.9±12.68 | ischemia  & hemorrhage | / | / | 2-16w | CIMT+CR | CR | ①⑤⑥ | / | Y |
| Dahl  2008  Norway | Norway | 30(18/12) | 23 | E:62±8 C:60±12 | ischemia  & hemorrhage | / | Y | 2w-8y | CIMT+CR | CR | ①③④⑦ | N | Y |
| Page  2008 | America | 35(13/12/10) | 36 | E:54.6±12.7 C1:60.75±14  C2:63.6±9.81 | ischemia  & hemorrhage | Y | / | 20-60m | CIMT+CR | CR  UC | ②③⑤ | N | / |
| Dromerick 2009 | America | 52(19/16/17) | 52 | E:62.8±12.8 C1:64.5±15.5  C2:64.7±14.6 | ischemia  & hemorrhage | / | Y | 1-28d | CIMT+CR | CR  UC | ④⑤ | / | / |
| Hammer 2009 | Sweden | 28(13/15) | 23 | E:66.3±10.3 C:60.4±11.1 | ischemia  & hemorrhage | / | / | 1-6m | CIMT | CR | ① | / | Y |
| Lin  2009 | China | 60(20/20/20) | 43 | E:55.28±9.34 C1:51.58±8.7  C2:50.7±13.9 | ischemia  & hemorrhage | / | Y | >6m | CIMT+CR | CR  UC | ①②④⑦ | / | / |
| Tariah  2010 | Jordan | 18(10/8) | 67 | E:54.8±10.9 C:60.6±4.6 | ischemia  & hemorrhage | Y | Y | 2.6-17.3m | CIMT | CR | ①②③ | / | Y |
| Wang  2011 | China | 30(10/10/10) | 47 | E:59.4±10.89 C1:63.5±9.63  C2:67±7.45 | ischemia  & hemorrhage | / | / | 11.3w | CIMT+CR | CR  UC | ③ | / | / |
| Khan  2011  China | China | 42(13/14/15) | 36 | E:60.4±16.1 C1:60.4±14.8  C2:62.2±13.5 | ischemia  & hemorrhage | / | / | 5.2-40.4m | CIMT+CR | CR  UC | ①③ | / | / |
| Huseyinsinoglu  2012 | Turkey | 22(11/11) | 45 | E:49.1±13.7 C:48.2±15.4 | ischemia  & hemorrhage | Y | / | 3-24m | CIMT+CR | CR | ①③④ | / | / |
| Smania 2012 | Italy | 68(39/29) | 17 | E:63.9±9.56 C:68.25±12.7 | ischemia  & hemorrhage | Y | / | 3-24m | CIMT | CR | ①③ | / | Y |
| Brunner 2012 | Norway | 30(14/16) | 37 | E:61±1 C:64.8±12.8 | ischemia  & hemorrhage | Y | / | 2-16w | CIMT+CR | CR | ①⑤⑥ | / | Y |
| Wu  2012 | China | 45(15/15/15) | 22 | E1:52.27±11.3  E2:54.87±10.2  C: 54.27±13 | ischemia  & hemorrhage | N | Y | E1:14.87±13.6m  E2:15±10.16m  C: 16.8±12.7m | CIMT+CR  CIMT | CR | ①② | / | / |
| Delden  2013 | Netherland | 60(22/19/19) | 32 | E:59.8±13.8 C1:62.6±9.8  C2:56.9±12.7 | ischemia  & hemorrhage | Y | / | 1-6m | CIMT+CR | CR  UC | ①②⑤⑥⑦ | / | Y |
| Hsieh  2014 | China | 48(16/16/16) | 29 | E:54.41±7.8 C1:52.34±13.2  C2: 54.12±10 | ischemia  & hemorrhage | / | Y | E:20.56±14m C1:23.56±15.4m  C2:27.81±19m | CIMT+CR | CR  UC | ①②③ | N | / |
| Masahiro  2014 | Japan | 66(44/22) | 42 | E:57.7±12.7 C:60.3±10.6 | ischemia  & hemorrhage | Y | Y | E:62.1±47.7m C:68±53.1m | CIMT | CR | ②③ | / | / |
| Yoon  2014 | Korea | 26(8/9/9) | 38 | E1:47.36±14.4  E2:64.33±8.5  C: 60.56±16.9 | ischemia  & hemorrhage | Y | / | E1:24.25±11.5d  E2:19.33±9.2d  C:24.78±11.6d | CIMT+CR  CIMT | CR | ②③ | / | / |
| El-Helow 2015 | Egypt | 60(30/30) | 35 | E:53.9±7.26 C: 52.7±9.87 | ischemia | Y | / | E:8.21±1.59d C:8.6±2.71d | CIMT | CR | ②⑤ | / | / |
| Barzel  2015 | Germany | 156(85/71) | 40 | E:62.55±13.7 C:66.3±12.63 | ischemia  & hemorrhage | / | / | >6m | CIMT+CR | CR | ①③⑥⑦ | N | Y |
| Thrane  2015 | Norway | 44(22/22) | 23 | E:65.3±8 C:61±14.8 | ischemia  & hemorrhage | N | Y | 7-32d | CIMT+CR | CR | ②③⑥ | Y | Y |
| Batool 2015 | Pakistan | 42(21/21) | 33 | E:49.67±7.01 C:49.47±8.19 | ischemia  & hemorrhage | Y | / | 1-14d | CIMT+CR | CR | ④ | / | / |
| Yadav  2016 | India | 60(30/30) | 27 | E:47.03±13.8 C:46.3±13.6 | ischemia  & hemorrhage | / | / | E:10.07±6.21m C:10.18±6.17m | CIMT | CR | ①② | N | Y |
| Kwakkel 2016 | Netherland | 58(29/29) | 47 | E:58.97±14 C:65.34±11.4 | ischemia | Y | / | 1-14d | CIMT+CR | CR | ①②③⑤⑥⑦ | N | Y |
| Treger  2016 | Israel | 28(9/19) | 29 | E:62±10.4 C:61.5±8.4 | ischemia | Y | / | E:39.8±28.4d C:23.3±24.1d | CIMT | CR | ④ | / | / |
| Liu  2016 | China | 90(31/32/27) | 44 | E1:67.87±9.4  E2:65.07±6.7  C: 66.8±6.11 | ischemia | Y | / | E1:9.69±1d  E2:8.44±0.6d  C:9.15±1.3d | CIMT+CR  CIMT | CR | ①②⑤ | / | Y |
| Seok  2016 | Korea | 30(10/10/10) | 43 | E:60.4±10.01 C1:58.7±12.37  C2: 62±11.8 | ischemia  & hemorrhage | Y | / | E:31.6±1.28d C1:38.2±14.34d  C2:32.3±16.51d | CIMT+CR | CR  UC | ②③ | N | Y |
| Yu  2017 | China | 29(15/14) | 24 | E:58.54±9.61 C:56.15±11.9 | ischemia | Y | / | 0-2w | CIMT+CR | CR | ①③ | / | Y |
| Gitendra 2018 | America | 21(10/11) | 14% | E:38.3-59.7 C:50.7-62.8 | ischemia  & hemorrhage | / | / | 5.1y | CIMT | CR | ①②③ | N | / |
| Baldwin 2018 | Australia | 19(10/9) | 32% | E:59.2±13.1 C:59.3±17 | ischemia  & hemorrhage | / | / | 28.85w | CIMT+CR | CR | ①③ | / | Y |
| Kim  2018 | Korea | 14(7/7) | / | / | ischemia  & hemorrhage | Y | Y | 0-3m | CIMT | CR | ① | / | / |
| Rocha  2021 | Brazil | 30(15/15) | 30 | E:59.66±10 C:59.8±9.59 | ischemia  & hemorrhage | / | / | >6m | CIMT | CR | ② | / | / |
| Hsieh  2021 | China | 35(13/12/10) | 66 | E1:55±5  E2:49±18.25  C: 57±7 | ischemia  & hemorrhage | / | / | E1:154±41.5d  E2:150.5±84d  C:123.5±74.3d | CIMT+CR  CIMT | CR | ②⑦ | / | Y |
| Takebayashi  2022 | Japan | 121(44/40/37) | 23 | E:59±12 C1:60±11  C2: 58±10 | ischemia  & hemorrhage | Y | / | E:37.6±55.8m C1:37.8±57.6m  C2:34.3±37.8m | CIMT+CR | CR  UC | ①② | N | / |
| CIMT for lower extremity motor dysfunction after stroke | | | | | | | | | | | | | |
| Zhu  2016 | China | 22(11/11) | 28 | E:59.18±7.34 C:58±6.97 | ischemia  & hemorrhage | Y | Y | 3-6m | CIMT+CR | CR | ⑧ | / | / |
| Choi  2017 | Korea | 24(12/12) | 42 | E:61.25±5.59 C:62.58±5.51 | ischemia  & hemorrhage | / | / | E:13.75±3.86d C:13.58±5.53d | CIMT+CR | CR | ⑧ | / | / |
| Silva  2017 | Brazil | 38(19/19) | 39 | 47-66 | ischemia  & hemorrhage | Y | Y | 0-12m | CIMT+CR | CR | ⑧ | N | Y |

Note: E: experimental group; C: control group; d:day; w:week; m:month; y:year; CIMT: constraint-induced movement therapy; CR: conventional rehabilitation; UC: usual care; /:not mentioned; Y:yes; N:no; ①a motor activity log for amount of use and quality of movement, MAL; ②fugl-myer assessment, FMA; ③the wolf motor function test, WMFT; ④function independent measure, FIM ⑤the action research-arm test, ARAT; ⑥nine hole peg Test, 9HPT ; ⑦stroke impact scale, SIS; ⑧gait parameters: step velocity, step length and step width.

**Table S3: The detailed information of CIMT**

| Included studies | Country | Type of intervention | Restriction time (per day) | Treatment period | Restriction position  (unaffected limbs) |
| --- | --- | --- | --- | --- | --- |
| CIMT for upper extremity motor dysfunction after stroke | | | | | |
| Dromerick  2000 | America | mCIMT | 6h | 14d | Y |
| Suputtitada  2004 | Thailand | CIMT | 6h | 10d | Y |
| Ploughman  2004 | Canada | CIMT | 2.7h | 14d | Y |
| Wolf  2006 | America | CIMT | 90% WH | 10d | Y |
| Wu  2007a | China | mCIMT | 6h | 15d | Y |
| Wu  2007b | China | mCIMT | 6h | 15d | Y |
| Boake  2007 | America | mCIMT | 90% WH | 12d | Y |
| Gauthier  2008 | America | mCIMT | 90% WH | 10d | Y |
| Myint  2008 | China | mCIMT | 90% WH | 10d | Y |
| Dahl  2008 | Norway | CIMT | 90% WH | 10d | Y |
| Page  2008 | America | mCIMT | 5h | 30d | Y |
| Dromerick  2009 | America | mCIMT | 6h | 10d | Y |
| Hammer  2009 | Sweden | CIMT | 6h | 10d | Y |
| Lin  2009 | China | mCIMT | 6h | 10d | Y |
| Tariah  2010 | Jordan | mCIMT | 2h | 60d | Y |
| Wang  2011 | China | mCIMT | 90% WH | 20d | Y |
| Khan  2011 | China | CIMT | 12h | 10d | Y |
| Huseyinsinoglu  2012 | Turkey | mCIMT | 90% WH | 10d | Y |
| Smania  2012 | Italy | mCIMT | 12h | 10d | Y |
| Brunner  2012 | Norway | CIMT | 4h | 28d | Y |
| Wu  2012 | China | mCIMT | 2h | 15d | Y |
| Delden  2013 | Netherland | CIMT | 6h | 30d | Y |
| Hsieh  2014 | China | mCIMT | 6h | 28d | Y |
| Masahiro  2014 | Japan | CIMT | 6h | 15d | Y |
| Yoon  2014 | Korea | CIMT | 6h | 10d | Y |
| El-Helow  2015 | Egypt | CIMT | 6h | 10d | Y |
| Barzel  2015 | Germany | mCIMT | 2h | 21d | Y |
| Thrane  2015 | Norway | mCIMT | 90% WH | 10d | Y |
| Batool  2015 | Pakistan | CIMT | 2h | 18d | Y |
| Yadav  2016 | India | mCIMT | 90% WH | 20d | Y |
| Kwakkel  2016 | Netherland | mCIMT | 3h | 15d | Y |
| Treger  2016 | Israel | mCIMT | 1h | 10d | Y |
| Liu  2016 | China | mCIMT | 4h | 10d |  |
| Seok  2016 | Korea | CIMT | 1h | 14d |  |
| Yu  2017 | China | mCIMT | 90% WH | 10d | Y |
| Gitendra  2018 | America | CIMT | 6h | 15d | Y |
| Baldwin  2018 | Australia | CIMT | 90% WH | 10d | Y |
| Kim  2018 | Korea | mCIMT | 6h | 10d | Y |
| Rocha  2021 | Brazil | CIMT | 1h | 24d | Y |
| Hsieh  2021 | China | mCIMT | 2h | 15d | Y |
| Takebayashi  2022 | Japan | CIMT | 1h | 30d | Y |
| CIMT for lower extremity motor dysfunction after stroke | | | | | |
| Zhu  2016 | China | mCIMT | 2h | 20d | Y |
| Choi  2017 | Korea | CIMT | 0.5h | 20d | Y |
| Silva  2017 | Brazil | CIMT | 0.5h | 10d | Y |

Note: h: hour; d: day; Y: yes; CIMT: constraint-induced movement therapy; mCIMT: modified constraint-induced movement therapy; WH: waking hours.

**Table S4: The overall reporting percentage of included studies in the CONSORT checklist**

| Included studies | Year | Number (n) | Percentage (%) |
| --- | --- | --- | --- |
| CIMT for upper extremity motor dysfunction after stroke | | | |
| Dromerick | 2000 | 20 | 54.1 |
| Suputtitada | 2004 | 18 | 48.6 |
| Ploughman | 2004 | 19 | 51.4 |
| Wolf | 2006 | 28 | 75.7 |
| Wu | 2007a | 20 | 54.1 |
| Wu | 2007b | 21 | 56.8 |
| Boake | 2007 | 20 | 54.1 |
| Gauthier | 2008 | 15 | 40.5 |
| Myint | 2008 | 20 | 54.1 |
| Dahl | 2008 | 22 | 59.5 |
| Page | 2008 | 19 | 51.4 |
| Dromerick | 2009 | 19 | 51.4 |
| Hammer | 2009 | 21 | 56.8 |
| Lin | 2009 | 21 | 56.8 |
| Tariah | 2010 | 22 | 59.5 |
| Wang | 2011 | 19 | 51.4 |
| Khan | 2011 | 22 | 59.5 |
| Huseyinsinoglu | 2012 | 23 | 62.2 |
| Smania | 2012 | 23 | 62.2 |
| Brunner | 2012 | 22 | 59.5 |
| Wu | 2012 | 22 | 59.5 |
| Delden | 2013 | 25 | 67.6 |
| Hsieh | 2014 | 25 | 67.6 |
| Masahiro | 2014 | 20 | 54.1 |
| Yoon | 2014 | 19 | 51.4 |
| El-Helow | 2015 | 17 | 45.9 |
| Barzel | 2015 | 27 | 73.0 |
| Thrane | 2015 | 23 | 62.2 |
| Batool | 2015 | 18 | 48.6 |
| Yadav | 2016 | 21 | 56.8 |
| Kwakkel | 2016 | 23 | 62.2 |
| Treger | 2016 | 21 | 56.8 |
| Liu | 2016 | 22 | 59.5 |
| Seok | 2016 | 19 | 51.4 |
| Yu | 2017 | 22 | 59.5 |
| Gitendra | 2018 | 19 | 51.4 |
| Baldwin | 2018 | 23 | 62.2 |
| Kim | 2018 | 14 | 37.8 |
| Rocha | 2021 | 17 | 45.9 |
| Hsieh | 2021 | 24 | 64.9 |
| Takebayashi | 2022 | 27 | 73.0 |
| CIMT for lower extremity motor dysfunction after stroke | | | |
| Zhu | 2016 | 18 | 48.6 |
| Choi | 2017 | 21 | 56.8 |
| Silva | 2017 | 24 | 64.9 |

**Table S5:** **The reporting quality of the CONSORT checklist**

| Section/Topic | Item No | Checklist item | Number (n) | Percentage (%) |
| --- | --- | --- | --- | --- |
| Title and abstract | 1a | Identification as a randomized study in the title | 23 | 52.2 |
|  | 1b | Structured summary of study design, methods, results, and conclusions (for specific guidance see CONSORT for abstracts) | 44 | 100.0 |
| Introduction | | | | |
| Background and objectives | 2a | Scientific background and explanation of rationale | 44 | 100.0 |
|  | 2b | Specific objectives or hypotheses | 44 | 100.0 |
| Methods | | | | |
| Study design | 3a | Description of study design (such as parallel, factorial) including allocation ratio | 22 | 50.0 |
|  | 3b | Important changes to methods after study commencement  (such as eligibility criteria), with reasons | 0 | 0 |
| Participants | 4a | Eligibility criteria for participants | 44 | 100.0 |
|  | 4b | Settings and locations where the data were collected | 36 | 81.8 |
| Interventions | 5 | The interventions for each group with sufficient details  to allow replication, including how and when they  were actually administered | 44 | 100.0 |
| Outcomes | 6a | Completely defined pre-specified primary and  secondary outcome measures, including how  and when they were assessed | 44 | 100.0 |
|  | 6b | Any changes to trial outcomes after the  trial commenced, with reasons | 0 | 0 |
| Sample size | 7a | How sample size was determined | 15 | 34.1 |
|  | 7b | When applicable, explanation of any  interim analyses and stopping guidelines | 0 | 0 |
| Randomization | | | | |
| Sequence generation | 8a | Method used to generate the random allocation sequence | 7 | 15.9 |
|  | 8b | Type of randomization; details of any restriction (such as blocking and block size) | 44 | 100.0 |
| Allocation concealment  mechanism | 9 | Mechanism used to implement the random allocation sequence (such as sequentially numbered containers), describing any steps taken to conceal the sequence until interventions were assigned | 14 | 31.8 |
| Implementation | 10 | Who generated the random allocation sequence, who enrolled participants, and who assigned participants to interventions | 0 | 0 |
| Blinding | 11a | If done, who was blinded after assignment to interventions (for example, participants, care providers, those assessing outcomes) and how | 31 | 70.5 |
|  | 11b | If relevant, description of the similarity of interventions | 30 | 68.2 |
| Statistical methods | 12a | Statistical methods used to compare groups for primary and secondary outcomes | 44 | 100.0 |
|  | 12b | Methods for additional analyses, such as subgroup analyses and adjusted analyses | 2 | 4.5 |
| Results | | | | |
| Participant flow | 13a | For each, the numbers of participants who were randomly assigned, received intended treatment, and were analyzed for the primary outcome group | 44 | 100.0 |
|  | 13b | For each group, losses and exclusions after randomization, together with reasons | 21 | 47.7 |
| Recruitment | 14a | Dates defining the periods of recruitment and follow-up | 44 | 100.0 |
|  | 14b | Why the trial ended or was stopped | 0 | 0 |
| Baseline data | 15 | A table showing baseline demographic and clinical characteristics for each group | 39 | 88.6 |
| Numbers analyzed | 16 | For each group, number of participants (denominator) included in each analysis and whether the analysis was by original assigned groups | 44 | 100.0 |
| Outcomes and estimation | 17a | For each primary and secondary outcome, results for each group, and the estimated effect size and its precision (such as 95% confidence interval) | 44 | 100.0 |
|  | 17b | For binary outcomes, presentation of both absolute and relative effect sizes is recommended | 0 | 0 |
| Ancillary analyses | 18 | Results of any other analyses performed, including subgroup analyses and adjusted analyses, distinguishing pre-specified from exploratory | 2 | 4.5 |
| Harms | 19 | All adverse harms or unintended effects in each group (for specific guidance see CONSORT for harms) | 5 | 11.4 |
| Discussion | | | | |
| Limitations | 20 | Study limitations, addressing sources of potential bias, imprecision, and, if relevant, multiplicity of analyzes | 32 | 72.7 |
| Generalizability | 21 | Generalizability (external validity, applicability) of the trial findings | 44 | 100.0 |
| Interpretation | 22 | Interpretation consistent with results, balancing benefits and harms, and considering other relevant evidence | 44 | 100.0 |
| Other information | | | | |
| Registration | 23 | Registration number and name of trial registry | 8 | 18.2 |
| Protocol | 24 | Where the full trial protocol can be accessed, if available | 8 | 18.2 |
| Funding | 25 | Sources of funding and other support (such as supply of drugs), role of funders | 16 | 36.4 |

**Table S6: Subgroup Analysis of CIMT plus CR vs. CR and CIMT vs. CR in MAL**

| **CIMT plus CR vs. CR** | | | | | | | | | |
| --- | --- | --- | --- | --- | --- | --- | --- | --- | --- |
|  | | **MAL-AOU** | | | | **MAL-QOM** | | | |
| **Subgroups** | **No. Of studies** | | **Effect Size**  **(MD, 95% Cl)** | ***P* value** | ***I*^2^** | **No. Of studies** | **Effect Size**  **(MD, 95% Cl)** | ***P* value** | ***I*^2^** |
| **Restricted time of CIMT daily** |  | |  |  |  |  |  |  |  |
| **1-5 hours** | 5 | | 0.45[−0.18, 1.09] | 0.16 | 80% | 5 | 0.52[−0.20, 1.25] | 0.16 | 84% |
| **6 hours** | 6 | | 0.34[0.02, 0.67] | 0.04 | 43% | 6 | 0.35[0.01, 0.71] | 0.04 | 52% |
| **>6 hours** | 7 | | 0.51[0.19, 0.83] | 0.002 | 48% | 7 | 0.64[0.49, 0.79] | <0.00001 | 0 |
| **Total restricted days of CIMT** |  | |  |  |  |  |  |  |  |
| **1-10 days** | 9 | | 0.66[0.29, 1.03] | 0.0005 | 76% | 9 | 0.74[0.45, 1.04] | <0.00001 | 57% |
| **11-20 days** | 4 | | 0.55[0.12, 0.99] | 0.01 | 25% | 4 | 0.57[0.21, 0.92] | 0.002 | 6% |
| **>20 days** | 5 | | 0.10[−0.14, 0.34] | 0.40 | 0 | 5 | 0.07[−0.24, 0.38] | 0.65 | 35% |
| **The stroke stage** |  | |  |  |  |  |  |  |  |
| **acute and subacute** | 9 | | 0.55[0.17, 0.93] | 0.004 | 77% | 9 | 0.62[0.27, 0.97] | 0.0005 | 73% |
| **chronic** | 9 | | 0.33[0.10, 0.56] | 0.006 | 20% | 9 | 0.36[0.11, 0.61] | 0.005 | 29% |
| **The types of CIMT** |  | |  |  |  |  |  |  |  |
| **mCIMT** | 11 | | 0.58[0.23, 0.93] | 0.001 | 70% | 11 | 0.61[0.26, 0.96] | 0.0007 | 73% |
| **CIMT** | 7 | | 0.24[0.17, 0.31] | <0.00001 | 0 | 7 | 0.43[0.16, 0.70] | 0.002 | 35% |
| **Follow-up time** |  | |  |  |  |  |  |  |  |
| **Immediately** | 18 | | 0.46[0.25, 0.67] | <0.0001 | 62% | 18 | 0.51[0.28, 0.73] | <0.00001 | 64% |
| **1-3 months after-treatment** | 8 | | 0.41[0.05, 0.77] | 0.04 | 52% | 8 | 0.27[0.07, 0.47] | 0.14 | 37% |
| **4-6 months after-treatment** | 2 | | -0.13[-0.53, 0.27] | 0.51 | 0 | 2 | -0.10[-0.48, 0.28] | 0.61 | 0 |
| **12 months after-treatment** | 1 | | 0.13[-0.07, 0.33] | 0.20 | / | 1 | 0.13[-0.07, 0.33] | 0.21 | / |
| **CIMT vs. CR** | | | | | | | | | |
|  | | **MAL-AOU** | | | | **MAL-QOM** | | | |
| **Subgroups** | **No. Of studies** | | **Effect Size (95% Cl)** | ***P* value** | ***I*^2^** | **No. Of studies** | **Effect Size (95% Cl)** | ***P* value** | ***I*^2^** |
| **Restricted time of CIMT daily** |  | |  |  |  |  |  |  |  |
| **1-5 hours** | 3 | | 0.26[-0.12, 0.64] | 0.18 | 35% | 3 | 0.42[-0.22, 1.06] | 0.20 | 55% |
| **6 hours** | 3 | | 0.29[0.13, 0.45] | 0.0005 | 0 | 3 | 0.26[0.12, 0.40] | 0.0002 | 0 |
| **>6 hours** | 4 | | 0.44[0.17, 0.71] | 0.002 | 0 | 4 | 0.75[0.10, 1.41] | 0.02 | 68% |
| **Total restricted days of CIMT** |  | |  |  |  |  |  |  |  |
| **1-10 days** | 5 | | 0.28[0.06, 0.50] | 0.01 | 0 | 5 | 0.35[0.13, 0.57] | 0.002 | 65% |
| **11-20 days** | 4 | | 0.34[0.17, 0.50] | <0.0001 | 3% | 4 | 0.31[0.17, 0.45] | <0.0001 | 42% |
| **>20 days** | 1 | | 0.51[-0.48, 1.50] | 0.31 | / | 1 | 0.58[-0.21, 1.37] | 0.15 | / |
| **The stroke stage** |  | |  |  |  |  |  |  |  |
| **acute and subacute** | 6 | | 0.24[0.01, 0.48] | 0.04 | 0 | 6 | 0.25[0.03, 0.48] | 0.03 | 0 |
| **chronic** | 4 | | 0.35[0.20, 0.51] | <0.0001 | 0 | 4 | 0.71[0.17, 1.25] | 0.01 | 75% |
| **The types of CIMT** |  | |  |  |  |  |  |  |  |
| **mCIMT** | 8 | | 0.37[0.15, 0.58] | 0.0009 | 0 | 8 | 0.55[0.18, 0.93] | 0.004 | 52% |
| **CIMT** | 2 | | 0.29[0.13, 0.46] | 0.0005 | 0 | 2 | 0.26[0.12, 0.40] | 0.0002 | 0 |
| **Follow-up time** |  | |  |  |  |  |  |  |  |
| **Immediately** | 10 | | 0.32[0.19, 0.45] | <0.00001 | 0 | 10 | 0.32[0.21, 0.44] | <0.00001 | 47% |
| **1-3 months after-treatment** | 6 | | 0.15[-0.41, 0.72] | 0.60 | 68% | 6 | 0.19[-0.10, 0.48] | 0.20 | 25% |
| **4-6 months after-treatment** | 1 | | 0.71[-0.03, 1.45] | 0.06 | / | 1 | 0.67[-0.40, 1.75] | 0.22 | / |

Note: No. of studies: number of studies; MAL-AOU: a motor activity log for amount of use; MAL-QOM: a motor activity log for quality of movement; 95% CI: 95% confidence interval

**Table S7: Meta-analysis of secondary outcomes**

| **Outcomes** | **No. Of studies** | **Effect Size (95% CI)** | ***P* value** | ***I*^2^** |
| --- | --- | --- | --- | --- |
| **CIMT combined CR vs. CR** | | | | |
| **FMA-UE** | 14 | MD: 2.42[1.05, 3.79] | 0.0005 | 0 |
| **WMFT-FA** | 14 | MD: 0.32[0.12, 0.51] | 0.001 | 50% |
| **ARAT** | 8 | MD: 6.41[4.49, 8.33] | <0.00001 | 0 |
| **FIM** | 11 | SMD: 0.66[-0.16, 1.48] | 0.11 | 87% |
| **9HPT** | 6 | MD: −0.01[0.05, 0.02] | 0.53 | 2% |
| **SIS-HF** | 8 | MD: 0.49[-1.78, 2.76] | 0.67 | 8% |
| **Gait** **parameters** | | | | |
| **SV** | 3 | MD: 0.10[0.01, 0.18] | 0.03 | 0 |
| **SL** | 3 | MD: 0.09[0.03, 0.14] | 0.001 | 0 |
| **SW** | 3 | MD: 0.03[0.01, 0.05] | 0.002 | 0 |
| **CIMT vs. CR** | | | | |
| **FMA-UE** | 11 | MD: 2.98[1.58, 4.37] | <0.0001 | 41% |
| **WMFT-FA** | 6 | MD: 0.42[0.18, 0.65] | 0.0005 | 0 |
| **ARAT** | 4 | MD: 9.82[3.70, 15.94] | 0.002 | 81% |
| **FIM** | 2 | SMD: 1.07[-1.53, 3.67] | 0.42 | 93% |
| **CIMT combined with CR vs. UC** | | | | |
| **FMA-UE** | 6 | MD: 3.64[1.87, 5.41] | <0.0001 | 3% |
| **WMFT-FA** | 5 | MD: 0.28[0.01, 0.58] | 0.02 | 17% |
| **ARAT** | 3 | MD: 8.12[5.70, 10.55] | <0.00001 | 21% |
| **FIM** | 2 | SMD: 0.83[-0.18, 3.49] | 0.06 | 88% |
| **SIS-HF** | 2 | MD: 9.26[-0.94, 19.46] | 0.08 | 0 |

Note: No. of studies: number of studies; MD: mean difference; SMD: standardized mean difference; CIMT: constraint induced movement therapy; CR: conventional rehabilitation; UC: usual care; 95%CI: 95% confidence interval; FMA-UE: Fugl-Myer Assessment of upper extremity; WMFT-FA: functional ability of the wolf motor function test; BI: Barthel Index; FIM: function independent measure; ARAT: the action research-arm test; 9HPT: nine hole peg Test; SIS-HF: hand function of stroke impact scale; SV: step velocity; SL: step length; SW: step width.
