# Supplementary material for: The effect and safety of constraint-induced movement therapy for post-stroke motor dysfunction: a meta-analysis and trial sequential analysis
Source: Front Neurol. 2023 Apr 18;14:1137320. doi: 10.3389/fneur.2023.1137320 (PMC10151521; doi:10.3389/fneur.2023.1137320)
Supplement: Supplementary file 2 [file Data_Sheet_2.ZIP › The reference lists of included studies.docx]

**The reference lists of included studies**

1. Dromerick AW, Edwards DF and Hahn M. Does the application of constraint-induced movement therapy during acute rehabilitation reduce arm impairment after ischemic stroke? *Stroke* 2000; 31: 2984-2988.

2. Suputtitada A, Suwanwela NC and Tumvitee S. Effectiveness of constraint-induced movement therapy in chronic stroke patients. *J Med Assoc Thai* 2004; 87: 1482-1490.

3. Ploughman M and Corbett D. Can forced-use therapy be clinically applied after stroke? An exploratory randomized controlled trial. *Arch Phys Med Rehabil* 2004; 85: 1417-1423.

4. Wolf SL, Winstein CJ, Miller JP, et al. Effect of constraint-induced movement therapy on upper extremity function 3 to 9 months after stroke: the EXCITE randomized clinical trial. *Jama* 2006; 296: 2095-2104.

5. Wu CY, Chen CL, Tang SF, et al. Kinematic and clinical analyses of upper-extremity movements after constraint-induced movement therapy in patients with stroke: a randomized controlled trial. *Arch Phys Med Rehabil* 2007; 88: 964-970.

6. Wu CY, Chen CL, Tsai WC, et al. A randomized controlled trial of modified constraint-induced movement therapy for elderly stroke survivors: changes in motor impairment, daily functioning, and quality of life. *Arch Phys Med Rehabil* 2007; 88: 273-278.

7. Boake C, Noser EA, Ro T, et al. Constraint-induced movement therapy during early stroke rehabilitation. *Neurorehabil Neural Repair* 2007; 21: 14-24.

8. Gauthier LV, Taub E, Perkins C, et al. Remodeling the brain: plastic structural brain changes produced by different motor therapies after stroke. *Stroke* 2008; 39: 1520-1525.

9. Myint JM, Yuen GF, Yu TK, et al. A study of constraint-induced movement therapy in subacute stroke patients in Hong Kong. *Clin Rehabil* 2008; 22: 112-124.

10. Dahl AE, Askim T, Stock R, et al. Short- and long-term outcome of constraint-induced movement therapy after stroke: a randomized controlled feasibility trial. *Clin Rehabil* 2008; 22: 436-447.

11. Page SJ, Levine P, Leonard A, et al. Modified constraint-induced therapy in chronic stroke: results of a single-blinded randomized controlled trial. *Phys Ther* 2008; 88: 333-340.

12. Dromerick AW, Lang CE, Birkenmeier RL, et al. Very Early Constraint-Induced Movement during Stroke Rehabilitation (VECTORS): A single-center RCT. *Neurology* 2009; 73: 195-201.

13. Hammer A and Lindmark B. Is forced use of the paretic upper limb beneficial? A randomized pilot study during subacute post-stroke recovery. *Clin Rehabil* 2009; 23: 424-433.

14. Lin KC, Chang YF, Wu CY, et al. Effects of constraint-induced therapy versus bilateral arm training on motor performance, daily functions, and quality of life in stroke survivors. *Neurorehabil Neural Repair* 2009; 23: 441-448.

15. Tariah HA, Almalty AM, Sbeih Z, et al. Constraint induced movement therapy for stroke survivors in Jordon a home-based model. *International Journal of Therapy and Rehabilitation* 2010; 17: 638-646.

16. Wang Q, Zhao JL, Zhu QX, et al. Comparison of conventional therapy, intensive therapy and modified constraint-induced movement therapy to improve upper extremity function after stroke. *J Rehabil Med* 2011; 43: 619-625.

17. Khan CM, Oesch PR, Gamper UN, et al. Potential effectiveness of three different treatment approaches to improve minimal to moderate arm and hand function after stroke--a pilot randomized clinical trial. *Clin Rehabil* 2011; 25: 1032-1041.

18. Huseyinsinoglu BE, Ozdincler AR and Krespi Y. Bobath Concept versus constraint-induced movement therapy to improve arm functional recovery in stroke patients: a randomized controlled trial. *Clin Rehabil* 2012; 26: 705-715.

19. Smania N, Gandolfi M, Paolucci S, et al. Reduced-intensity modified constraint-induced movement therapy versus conventional therapy for upper extremity rehabilitation after stroke: a multicenter trial. *Neurorehabil Neural Repair* 2012; 26: 1035-1045.

20. Brunner IC, Skouen JS and Strand LI. Is modified constraint-induced movement therapy more effective than bimanual training in improving arm motor function in the subacute phase post stroke? A randomized controlled trial. *Clin Rehabil* 2012; 26: 1078-1086.

21. Wu CY, Chen YA, Chen HC, et al. Pilot trial of distributed constraint-induced therapy with trunk restraint to improve poststroke reach to grasp and trunk kinematics. *Neurorehabil Neural Repair* 2012; 26: 247-255.

22. van Delden AL, Peper CL, Nienhuys KN, et al. Unilateral versus bilateral upper limb training after stroke: the Upper Limb Training After Stroke clinical trial. *Stroke* 2013; 44: 2613-2616.

23. Hsieh YW, Lin KC, Horng YS, et al. Sequential combination of robot-assisted therapy and constraint-induced therapy in stroke rehabilitation: a randomized controlled trial. *J Neurol* 2014; 261: 1037-1045.

24. Abo M, Kakuda W, Momosaki R, et al. Randomized, multicenter, comparative study of NEURO versus CIMT in poststroke patients with upper limb hemiparesis: the NEURO-VERIFY Study. *Int J Stroke* 2014; 9: 607-612.

25. Yoon JA, Koo BI, Shin MJ, et al. Effect of constraint-induced movement therapy and mirror therapy for patients with subacute stroke. *Ann Rehabil Med* 2014; 38: 458-466.

26. El-Helow MR, Zamzam ML, Fathalla MM, et al. Efficacy of modified constraint-induced movement therapy in acute stroke. *Eur J Phys Rehabil Med* 2015; 51: 371-379.

27. Barzel A, Ketels G, Stark A, et al. Home-based constraint-induced movement therapy for patients with upper limb dysfunction after stroke (HOMECIMT): a cluster-randomised, controlled trial. *Lancet Neurol* 2015; 14: 893-902.

28. Thrane G, Askim T, Stock R, et al. Efficacy of Constraint-Induced Movement Therapy in Early Stroke Rehabilitation: A Randomized Controlled Multisite Trial. *Neurorehabil Neural Repair* 2015; 29: 517-525.

29. Batool S, Soomro N, Amjad F, et al. To compare the effectiveness of constraint induced movement therapy versus motor relearning programme to improve motor function of hemiplegic upper extremity after stroke. *Pak J Med Sci* 2015; 31: 1167-1171.

30. Yadav RK, Sharma R, Borah D, et al. Efficacy of Modified Constraint Induced Movement Therapy in the Treatment of Hemiparetic Upper Limb in Stroke Patients: A Randomized Controlled Trial. *J Clin Diagn Res* 2016; 10: Yc01-yc05.

31. Kwakkel G, Winters C, van Wegen EE, et al. Effects of Unilateral Upper Limb Training in Two Distinct Prognostic Groups Early After Stroke: The EXPLICIT-Stroke Randomized Clinical Trial. *Neurorehabil Neural Repair* 2016; 30: 804-816.

32. Treger I, Aidinof L, Lehrer H, et al. Modified constraint-induced movement therapy improved upper limb function in subacute poststroke patients: a small-scale clinical trial. *Top Stroke Rehabil* 2012; 19: 287-293.

33. Liu KP, Balderi K, Leung TL, et al. A randomized controlled trial of self-regulated modified constraint-induced movement therapy in sub-acute stroke patients. *Eur J Neurol* 2016; 23: 1351-1360.

34. Seok H, Lee SY, Kim J, et al. Can Short-Term Constraint-Induced Movement Therapy Combined With Visual Biofeedback Training Improve Hemiplegic Upper Limb Function of Subacute Stroke Patients? *Ann Rehabil Med* 2016; 40: 998-1009.

35. Yu C, Wang W, Zhang Y, et al. The Effects of Modified Constraint-Induced Movement Therapy in Acute Subcortical Cerebral Infarction. *Front Hum Neurosci* 2017; 11: 265.

36. Uswatte G, Taub E, Bowman MH, et al. Rehabilitation of stroke patients with plegic hands: Randomized controlled trial of expanded Constraint-Induced Movement therapy. *Restor Neurol Neurosci* 2018; 36: 225-244.

37. Baldwin CR, Harry AJ, Power LJ, et al. Modified Constraint-Induced Movement Therapy is a feasible and potentially useful addition to the Community Rehabilitation tool kit after stroke: A pilot randomised control trial. *Aust Occup Ther J* 2018; 65: 503-511.

38. Kim JH and Chang MY. Effects of modified constraint-induced movement therapy on upper extremity function and occupational performance of stroke patients. *J Phys Ther Sci* 2018; 30: 1092-1094.

39. Rocha LSO, Gama GCB, Rocha RSB, et al. Constraint Induced Movement Therapy Increases Functionality and Quality of Life after Stroke. *J Stroke Cerebrovasc Dis* 2021; 30: 105774.

40. Hsieh HC, Liao RD, Yang TH, et al. The clinical effect of Kinesio taping and modified constraint-induced movement therapy on upper extremity function and spasticity in patients with stroke: a randomized controlled pilot study. *Eur J Phys Rehabil Med* 2021; 57: 511-519.

41. Takebayashi T, Takahashi K, Amano S, et al. Robot-Assisted Training as Self-Training for Upper-Limb Hemiplegia in Chronic Stroke: A Randomized Controlled Trial. *Stroke* 2022; 53: 2182-2191.

42. Zhu Y, Zhou C, Liu Y, et al. Effects of modified constraint-induced movement therapy on the lower extremities in patients with stroke: a pilot study. *Disabil Rehabil* 2016; 38: 1893-1899.

43. Choi HS, Shin WS, Bang DH, et al. Effects of Game-Based Constraint-Induced Movement Therapy on Balance in Patients with Stroke: A Single-Blind Randomized Controlled Trial. *Am J Phys Med Rehabil* 2017; 96: 184-190.

44. EMGS ES, Ribeiro TS, da Silva TCC, et al. Effects of constraint-induced movement therapy for lower limbs on measurements of functional mobility and postural balance in subjects with stroke: a randomized controlled trial. *Top Stroke Rehabil* 2017; 24: 555-561.
