# Supplementary figures and images for: The effect and safety of constraint-induced movement therapy for post-stroke motor dysfunction: a meta-analysis and trial sequential analysis
Source: Front Neurol. 2023 Apr 18;14:1137320. doi: 10.3389/fneur.2023.1137320 (PMC10151521; doi:10.3389/fneur.2023.1137320)

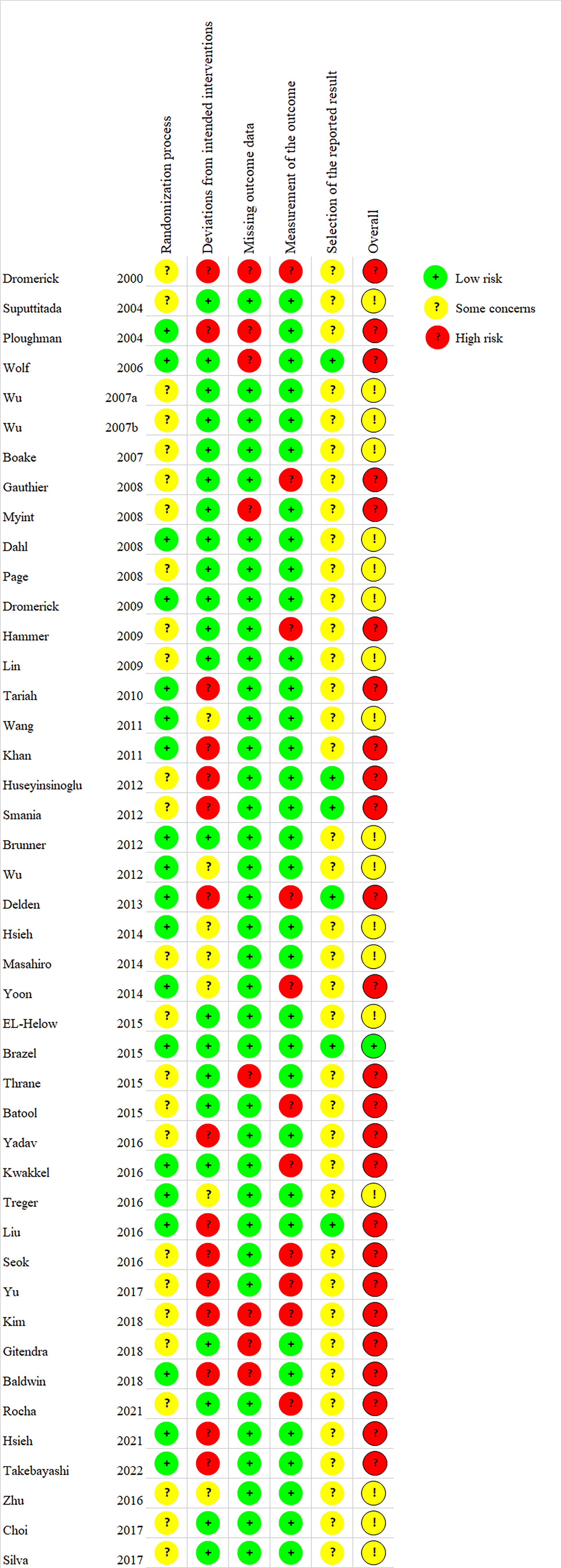

Supplement: Supplementary file 2 [file Data_Sheet_2.ZIP › Figure S1.tif]

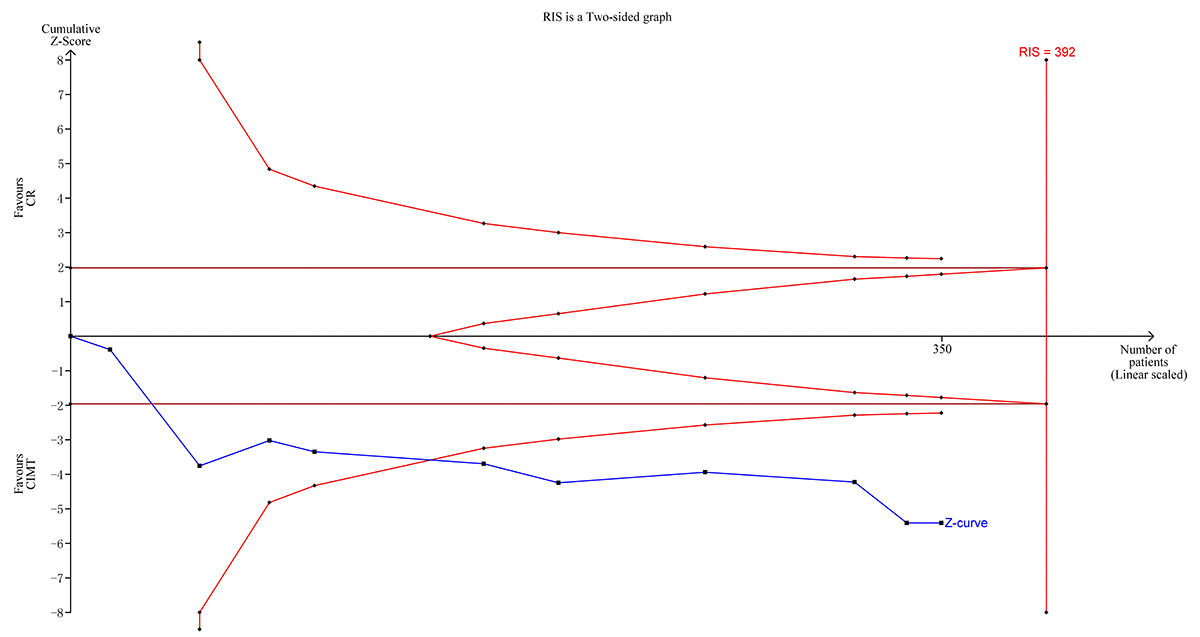

Supplement: Supplementary file 2 [file Data_Sheet_2.ZIP › Figure S10.tif]

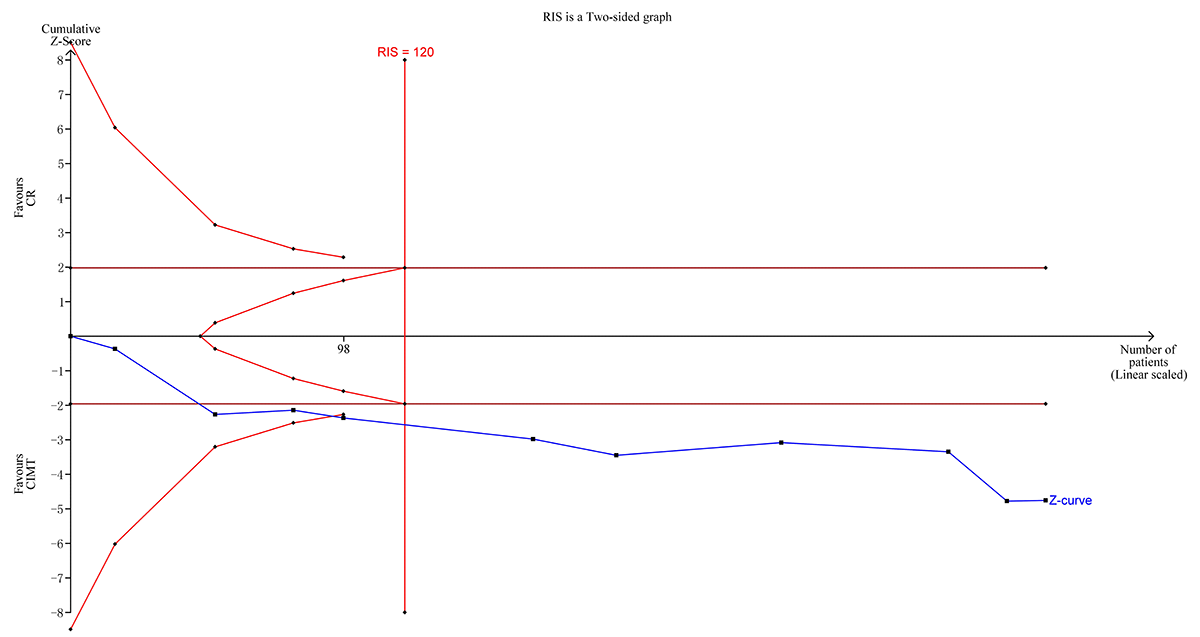

Supplement: Supplementary file 2 [file Data_Sheet_2.ZIP › Figure S11.tif]

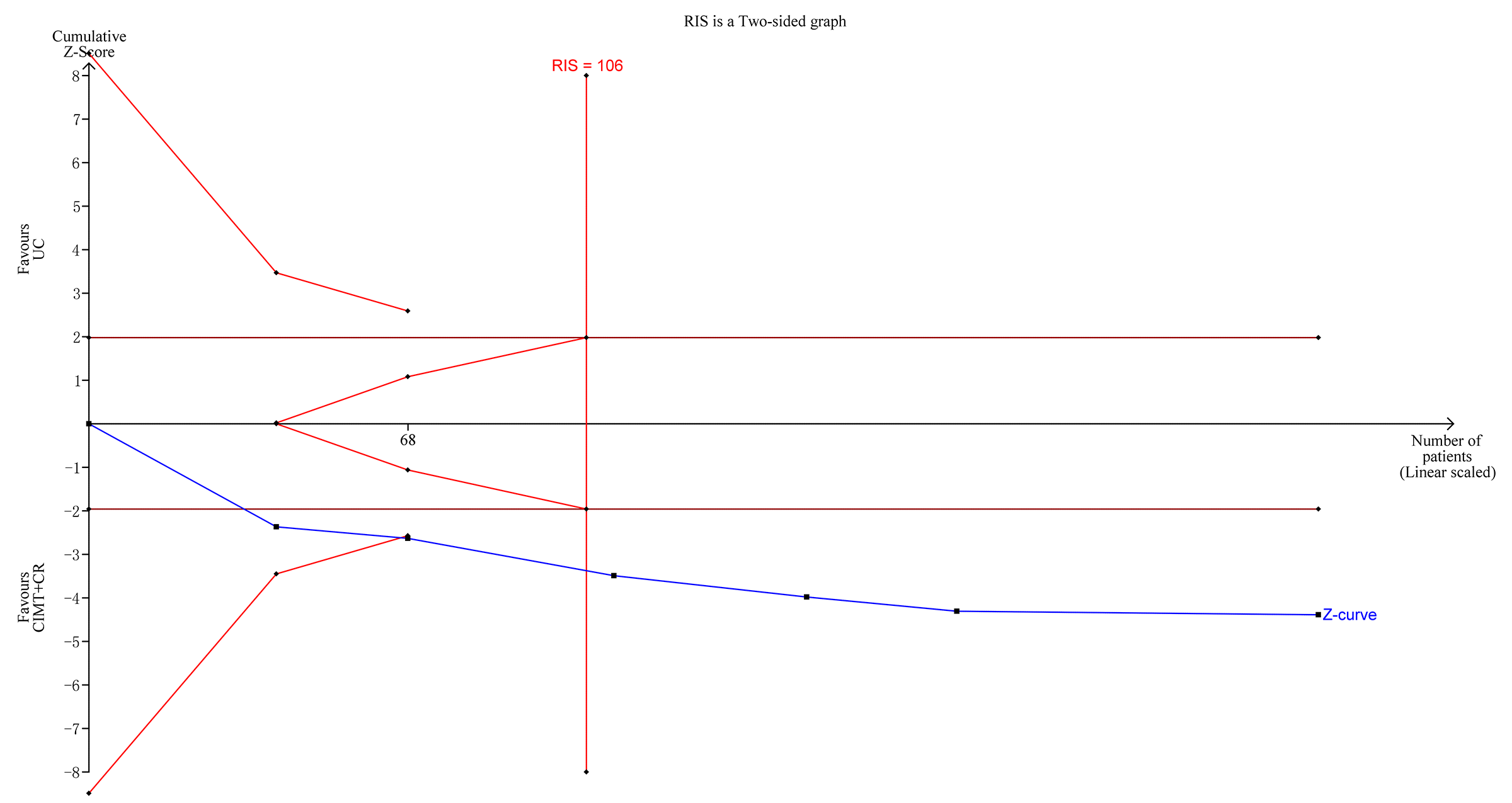

Supplement: Supplementary file 2 [file Data_Sheet_2.ZIP › Figure S12.tif]

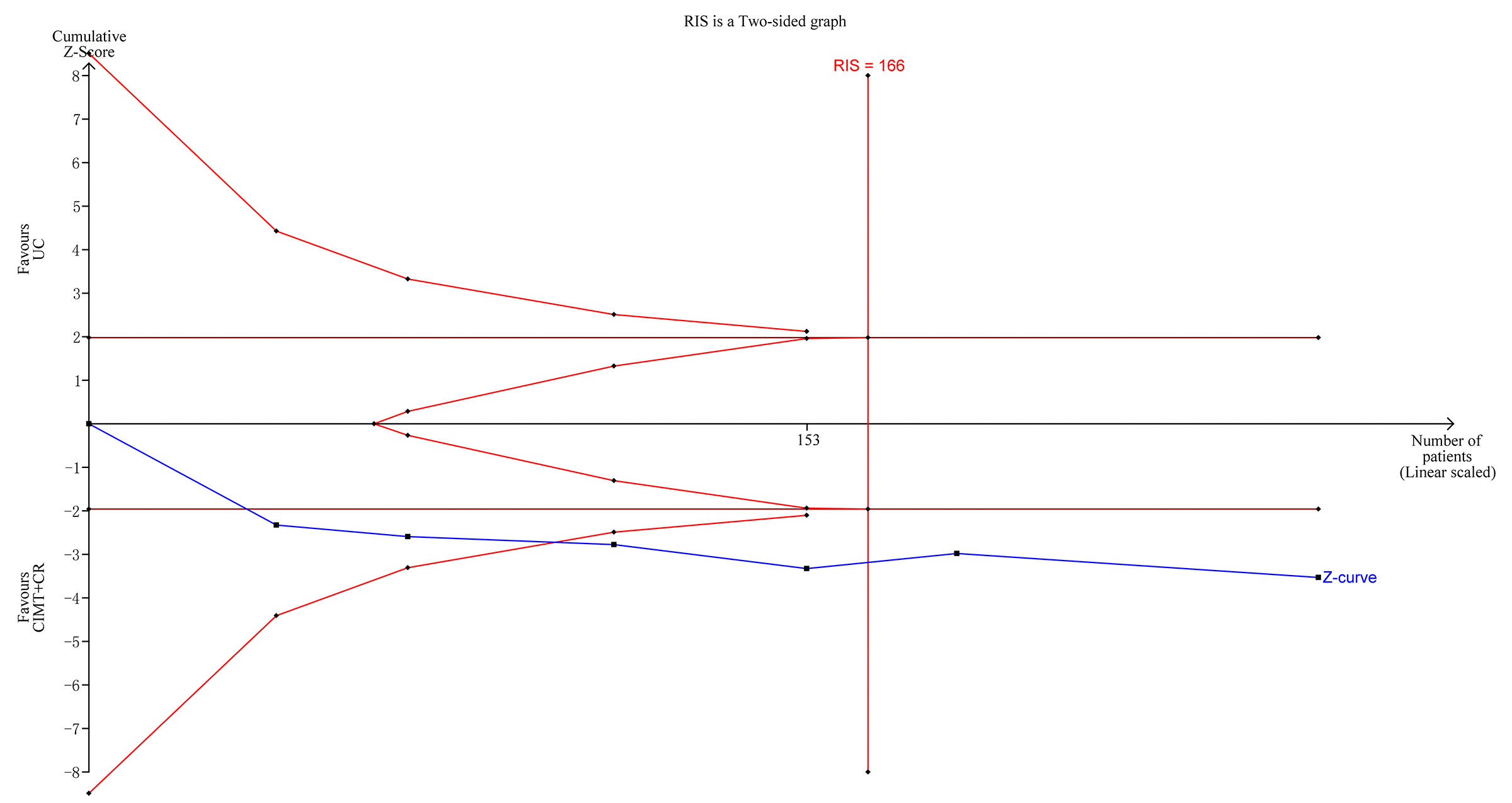

Supplement: Supplementary file 2 [file Data_Sheet_2.ZIP › Figure S13.tif]

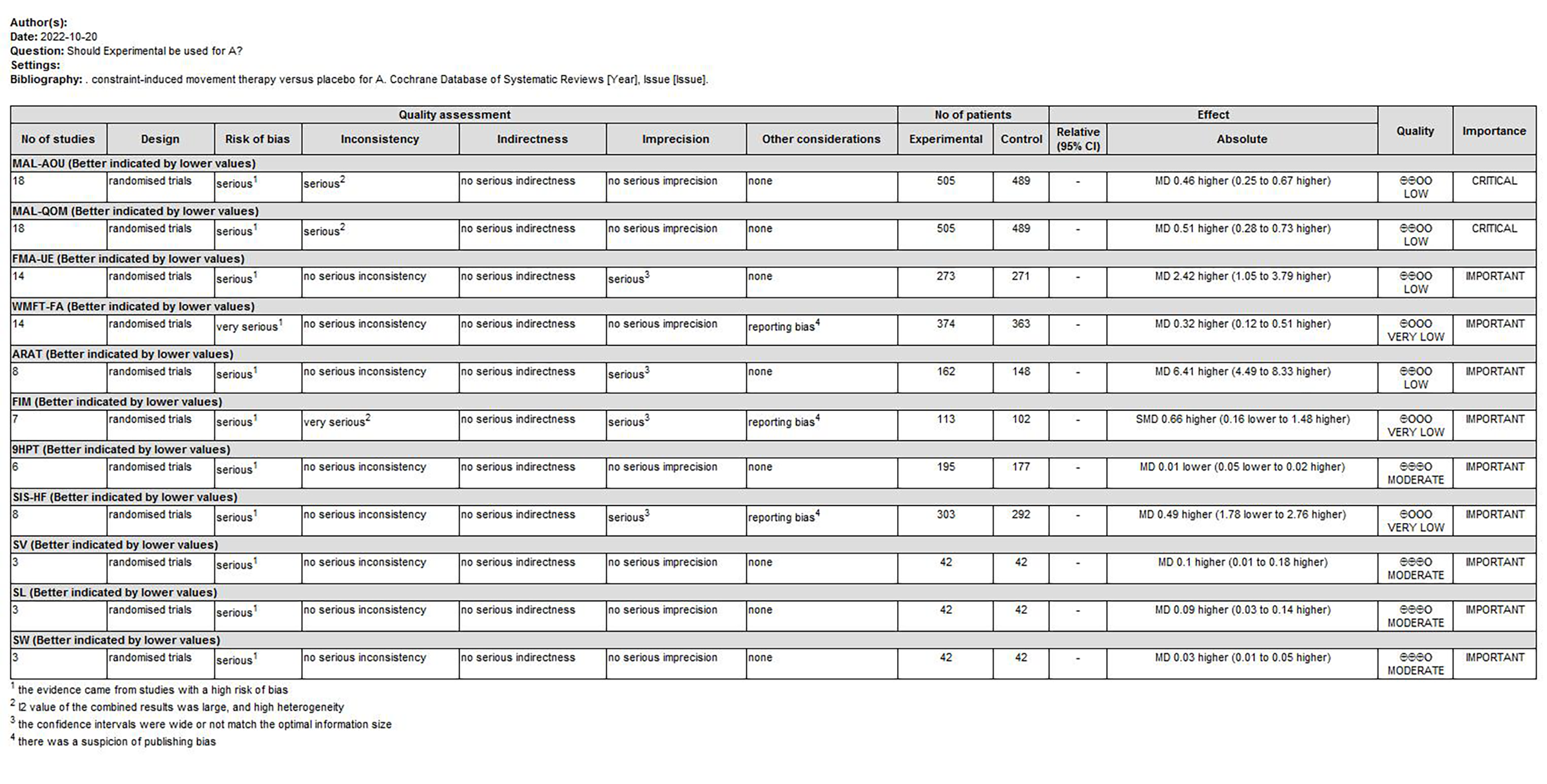

Supplement: Supplementary file 2 [file Data_Sheet_2.ZIP › Figure S14.tif]

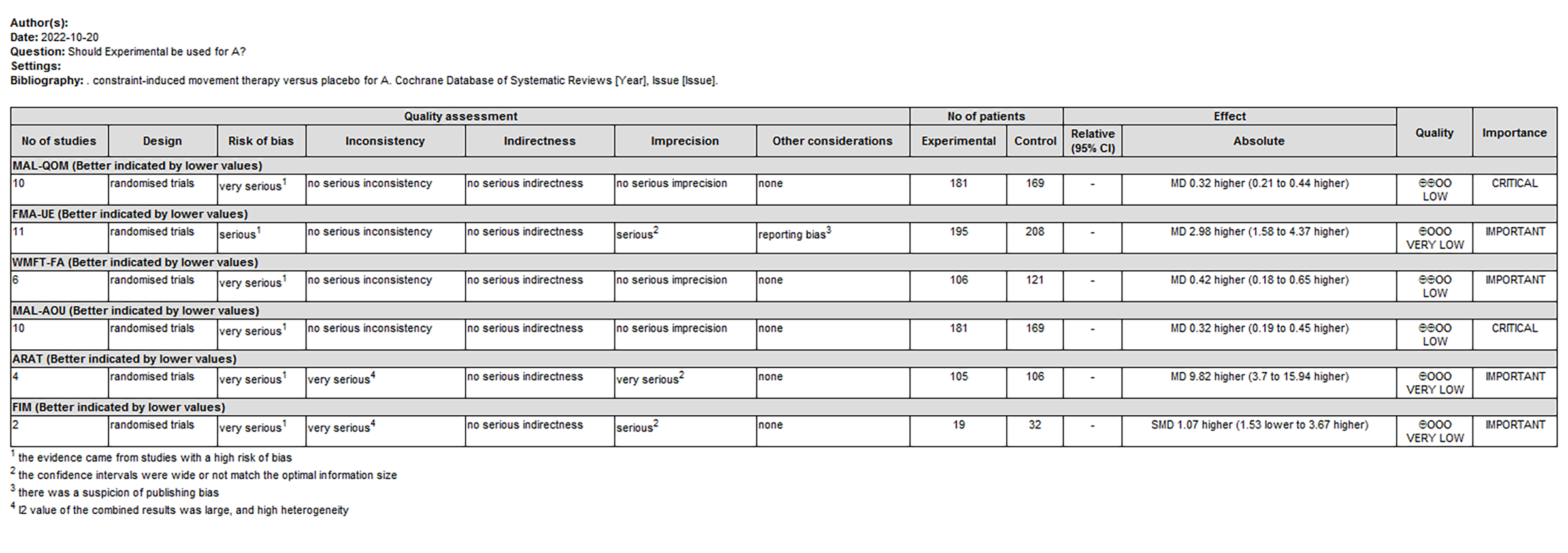

Supplement: Supplementary file 2 [file Data_Sheet_2.ZIP › Figure S15.tif]

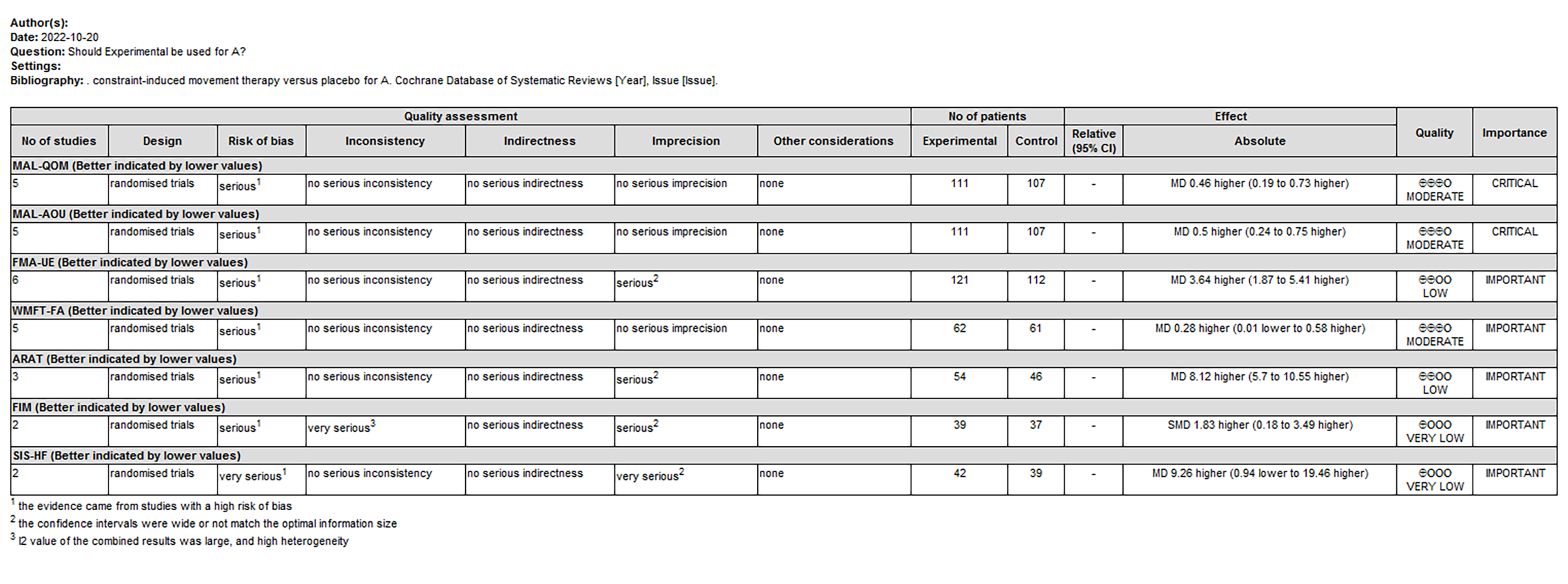

Supplement: Supplementary file 2 [file Data_Sheet_2.ZIP › Figure S16.tif]

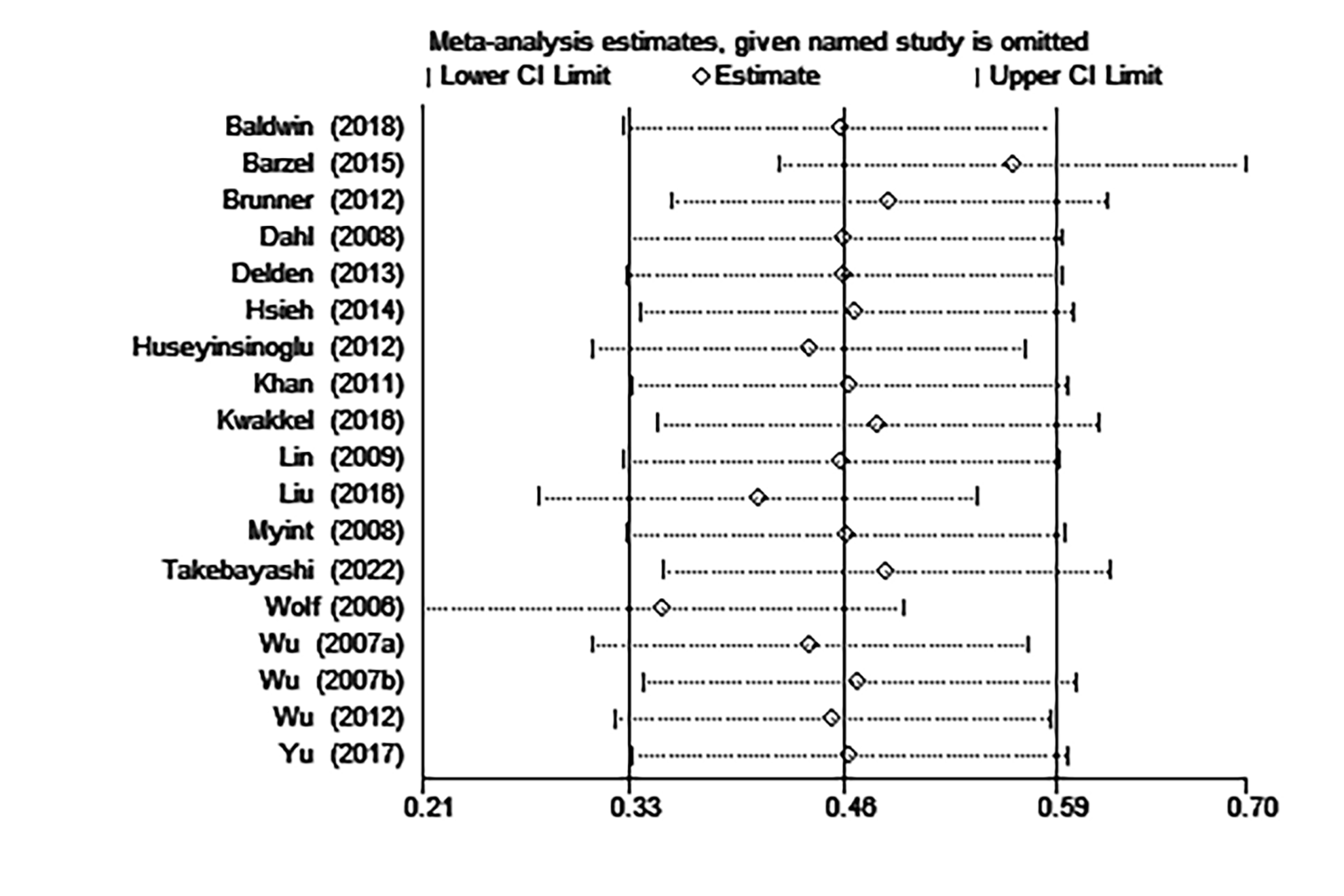

Supplement: Supplementary file 2 [file Data_Sheet_2.ZIP › Figure S2.tif]

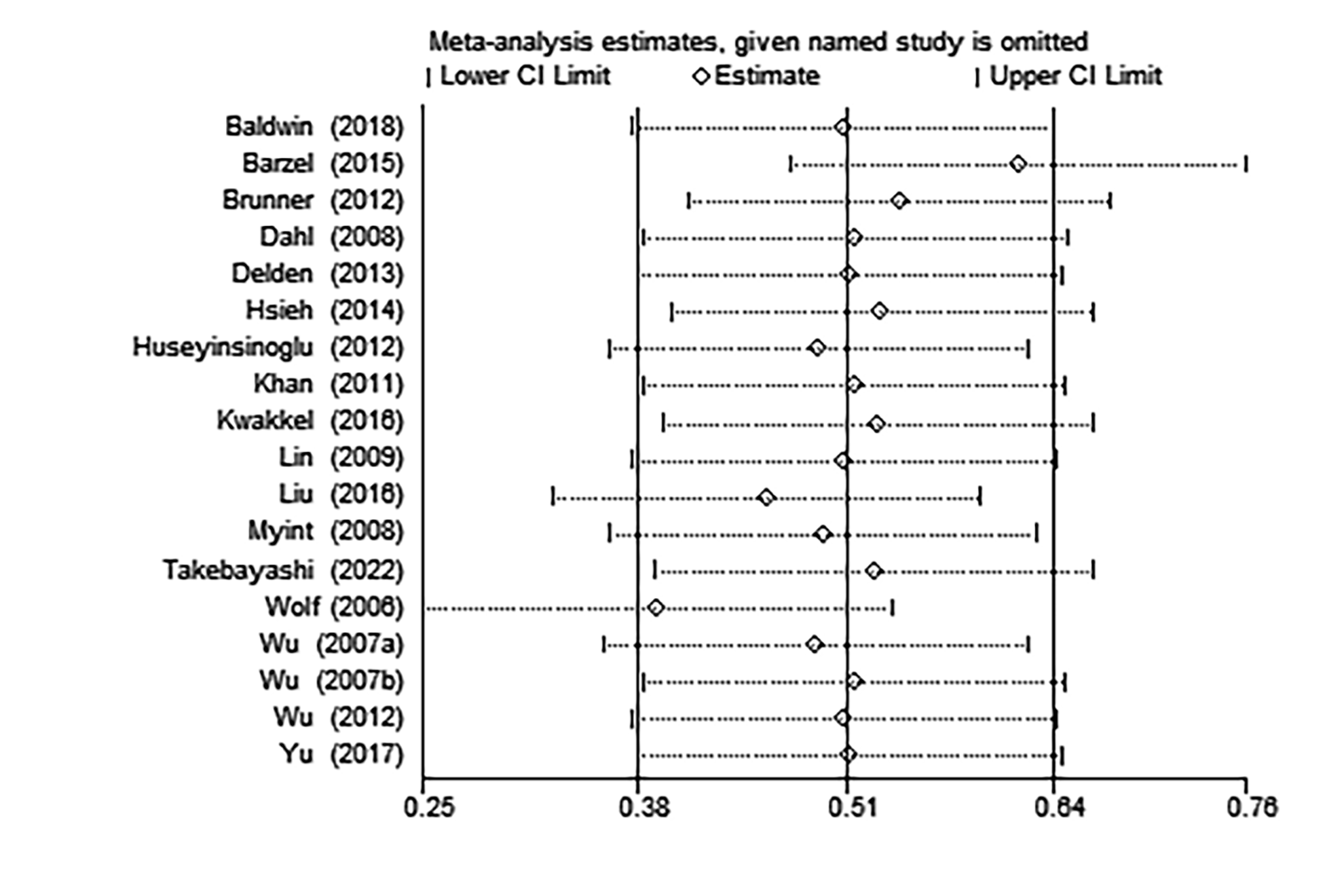

Supplement: Supplementary file 2 [file Data_Sheet_2.ZIP › Figure S3.tif]

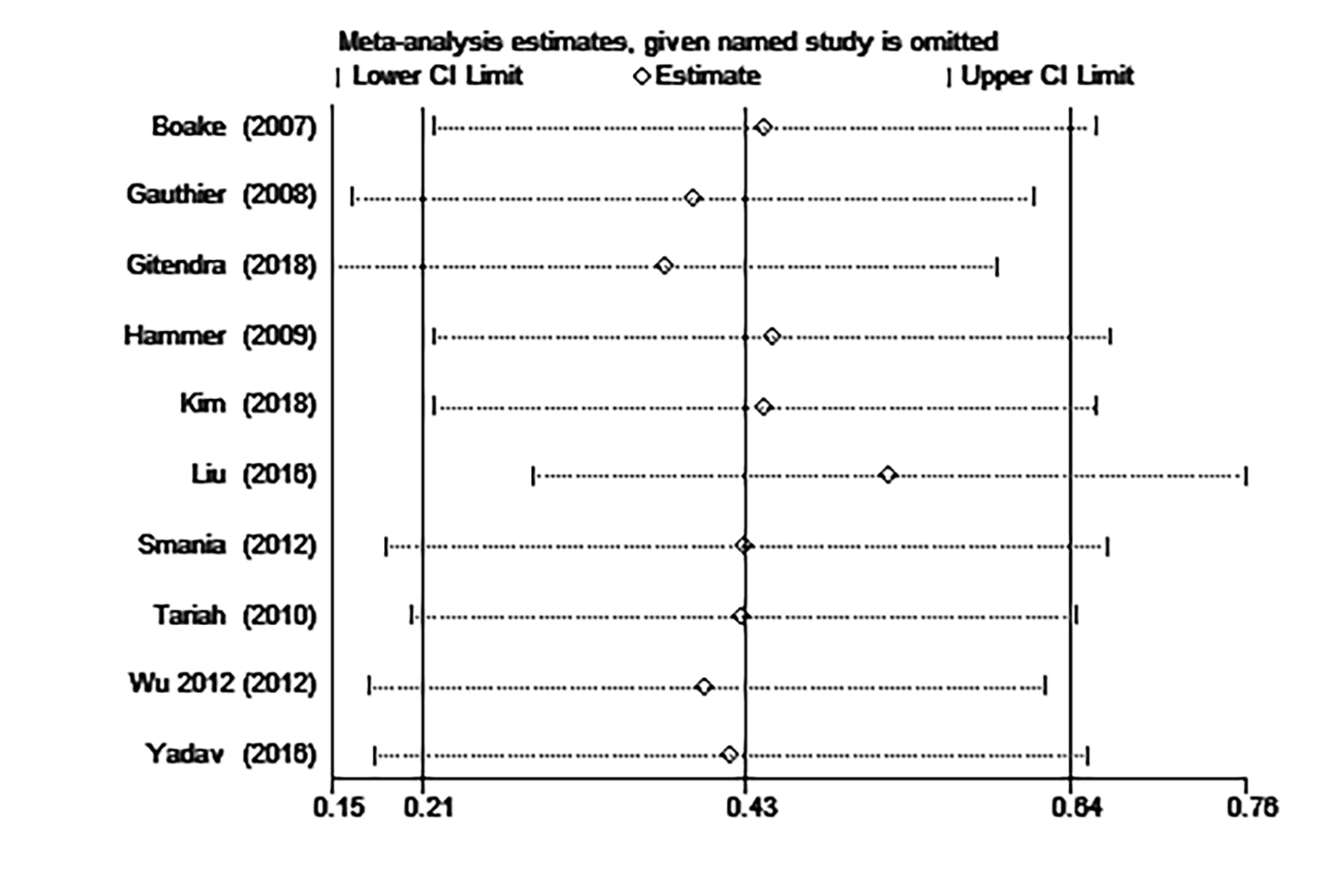

Supplement: Supplementary file 2 [file Data_Sheet_2.ZIP › Figure S4.tif]

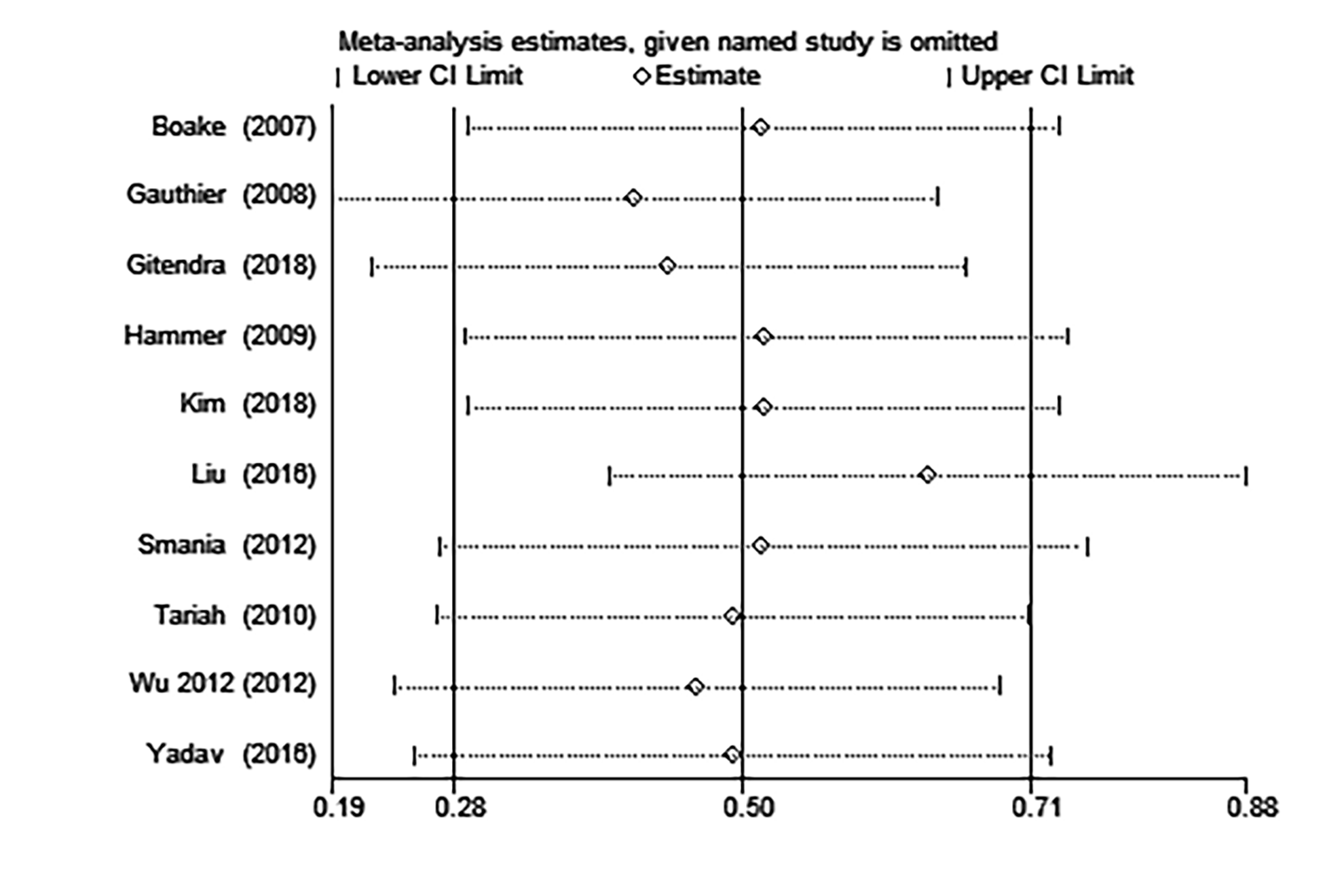

Supplement: Supplementary file 2 [file Data_Sheet_2.ZIP › Figure S5.tif]

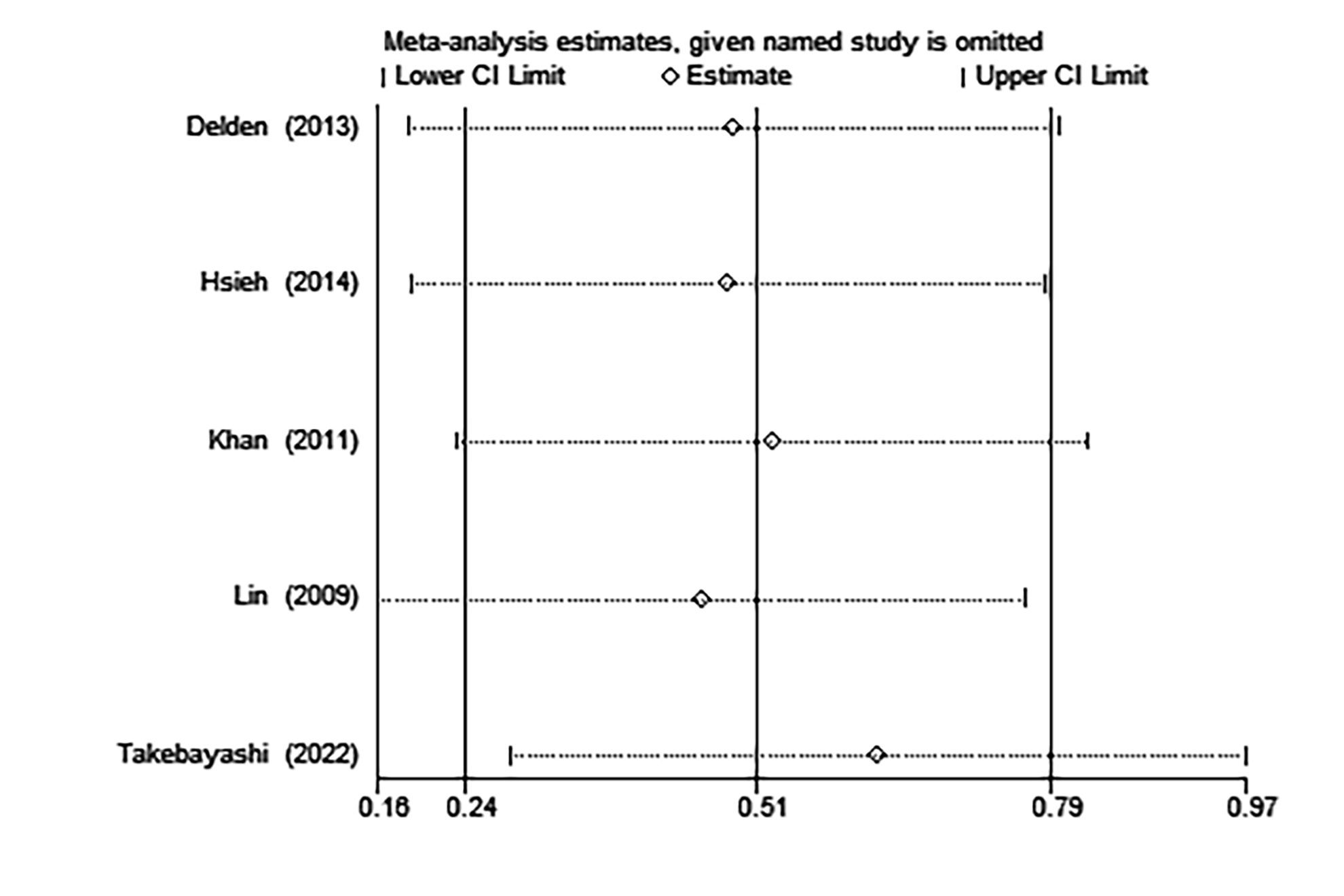

Supplement: Supplementary file 2 [file Data_Sheet_2.ZIP › Figure S6.tif]

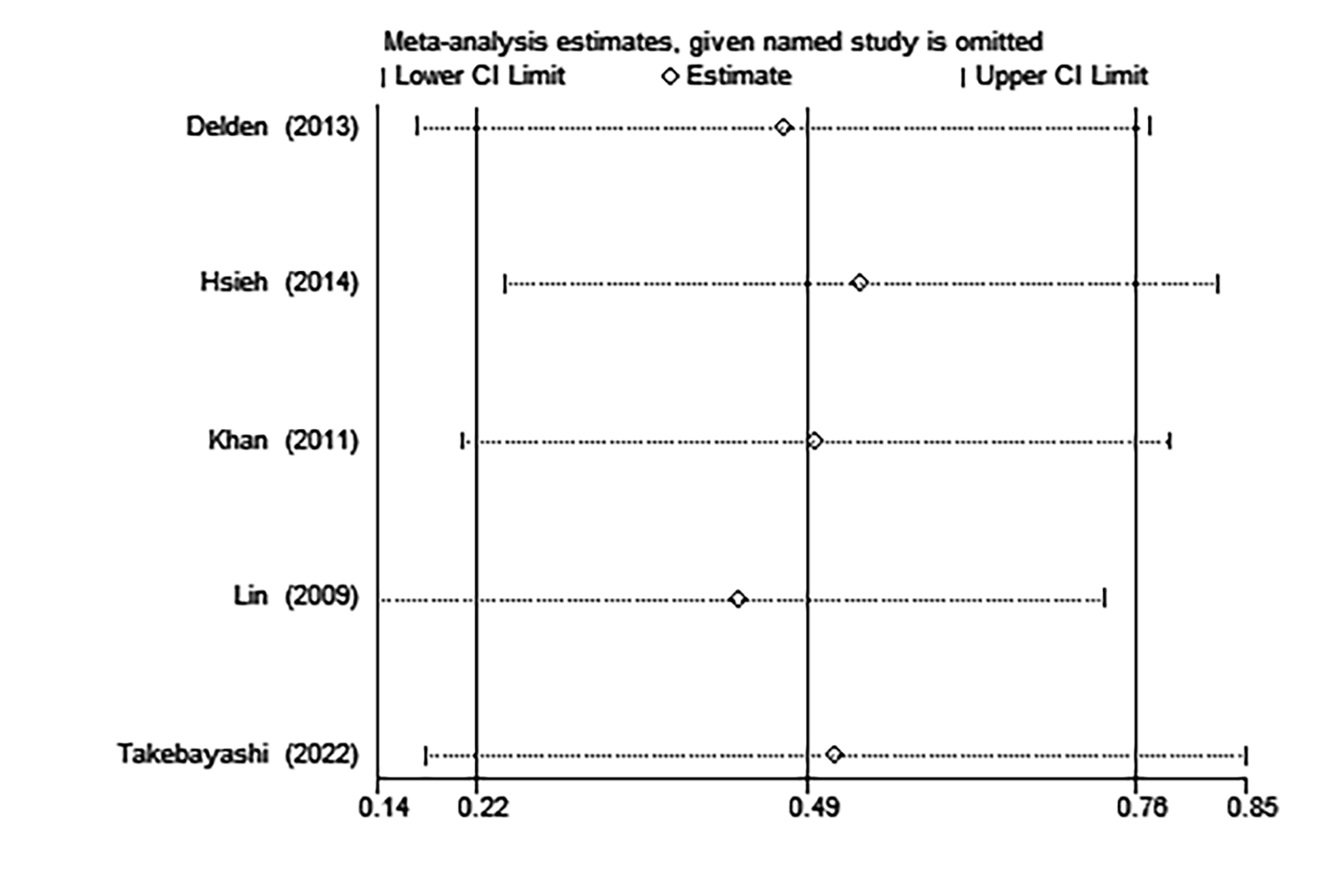

Supplement: Supplementary file 2 [file Data_Sheet_2.ZIP › Figure S7.tif]

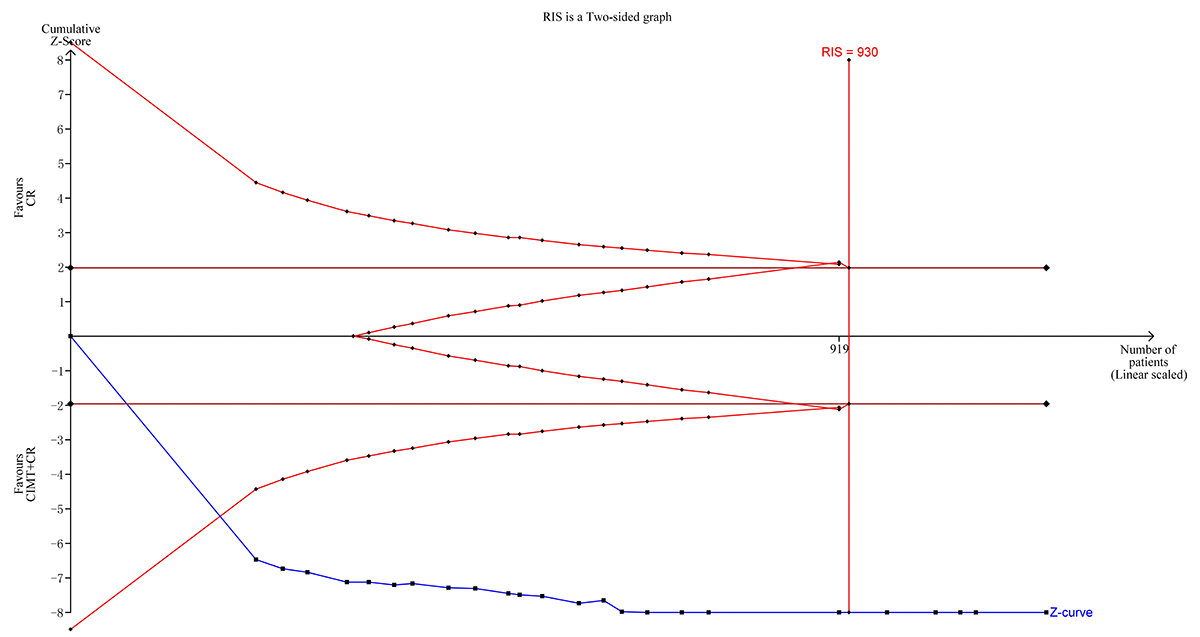

Supplement: Supplementary file 2 [file Data_Sheet_2.ZIP › Figure S8.tif]

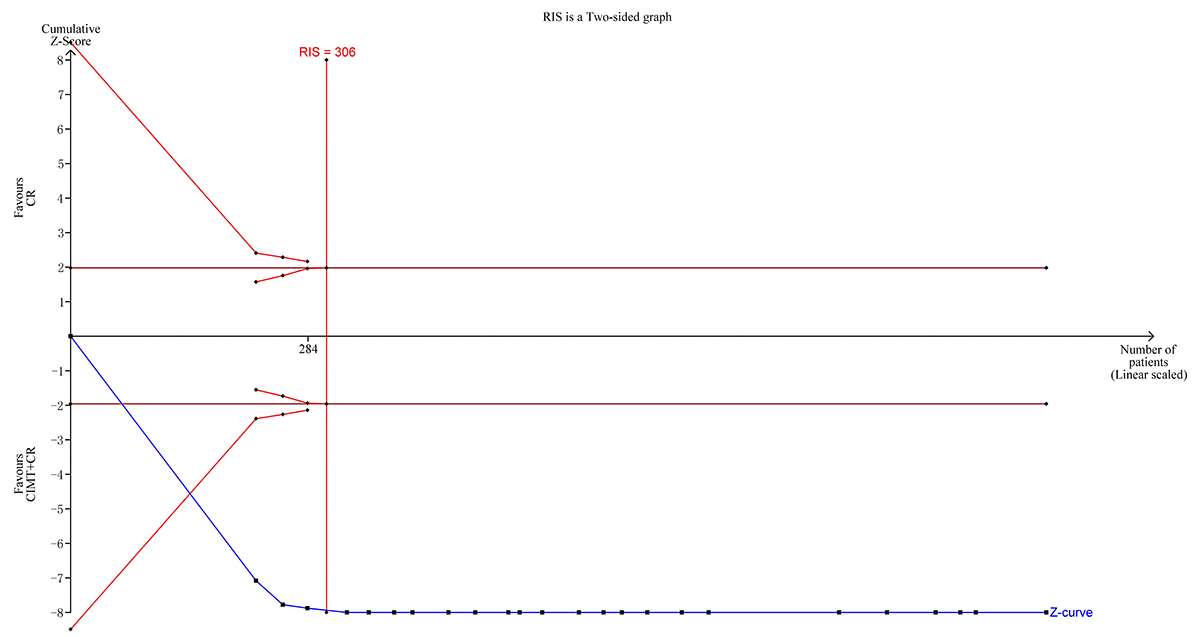

Supplement: Supplementary file 2 [file Data_Sheet_2.ZIP › Figure S9.tif]
