# Supplementary material for: The effect and safety of constraint-induced movement therapy for post-stroke motor dysfunction: a meta-analysis and trial sequential analysis
Source: Front Neurol. 2023 Apr 18;14:1137320. doi: 10.3389/fneur.2023.1137320 (PMC10151521; doi:10.3389/fneur.2023.1137320)
Supplement: Supplementary file 1 [file Data_Sheet_1.ZIP › Search Strategy.doc]

search strategy

PubMed search strategy

Search Date: 31st October 2022

1 " Movement Disorders "[MeSH Terms]

2 " Movement Disorder* " [Title/Abstract]

3 " Dyskinesia Syndrome " [Title/Abstract]

4 " Motor dysfunction " [Title/Abstract]

5 " Limb Dysfunction " [Title/Abstract]
6 " Upper Limb Dysfunction " [Title/Abstract]
7 " Lower Limb Dysfunction " [Title/Abstract]
8 " Extremity Dysfunction " [Title/Abstract]
9 " Limb " [Title/Abstract]
10 " Extremity " [Title/Abstract]
11 1 OR 2 OR 3 OR 4 OR 5 OR 6 OR 7 OR 8 OR 9 OR 10
12 " Stoke" [MeSH Terms]
13 " Stroke* " [Title/Abstract]
14 " Hemiplegia " [Title/Abstract]

15 " Cerebrovascular Accident* " [Title/Abstract]

16 " CVA* (Cerebrovascular Accident) " [Title/Abstract]

17 " Cerebrovascular Apoplexy " [Title/Abstract]

18 " Vascular Accident*, Brain" [Title/Abstract]

19 " Brain Vascular Accident*" [Title/Abstract]

20 " Cerebrovascular Stroke*" [Title/Abstract]

21 12 OR 13 OR 14 OR 15 OR 16 OR 17 OR 18 OR 19 OR 20

22 " Constraint-Induced Movement Therapy " [MeSH Terms]

23 " Constraint Induced Movement Therapy " [Title/Abstract]

24 " CIMT " [Title/Abstract]

25 " modified Constraint Induced Movement Therapy " [Title/Abstract]

26 " modified CIMT " [Title/Abstract]

27 " Constraint Induced Therapy " [Title/Abstract]

28 " Forced Use Therapy " [Title/Abstract]

29 22 OR 23 OR 24 OR 25 OR 26 OR 27 OR 28

30 11 AND 21 AND 29

EMBASE search strategy

Search Date: 31st October 2022

1 exp Movement Disorders/
2 Movement Disorder*.tw.
3 Dyskinesia Syndrome.tw.
4 Motor dysfunction.tw.
5 Limb Dysfunction.tw.

6 Upper Limb Dysfunction.tw.
7 Lower Limb Dysfunction.tw.
8 Extremity Dysfunction.tw.
9 Limb.tw.
10 Extremity.tw.
11 or/1-10
12 exp Stoke/
13 Stroke*.tw.

14 Hemiplegia.tw.
15 Cerebrovascular Accident*.tw.
16 CVA* (Cerebrovascular Accident).tw.
17 Cerebrovascular Apoplexy.tw.
18 Vascular Accident*, Brain.tw.
19 Brain Vascular Accident*.tw.
20 Cerebrovascular Stroke*.tw.
21 or/12-20
22 exp Constraint-Induced Movement Therapy/
23 Constraint Induced Movement Therapy.tw.
24 CIMT.tw.
25 modified Constraint Induced Movement Therapy.tw.
26 modified CIMT.tw.
27 Constraint Induced Therapy.tw.
28 Forced Use Therapy.tw.

29 or/22-28

30 11 and 21 and 29

Cochrane Library search strategy

Search Date: 31st October 2022

#1 " Movement Disorders "[MeSH Terms]

#2 " Movement Disorder* " [Title/Abstract]

#3 " Dyskinesia Syndrome " [Title/Abstract]

#4 " Motor dysfunction " [Title/Abstract]

#5 " Limb Dysfunction " [Title/Abstract]
#6 " Upper Limb Dysfunction " [Title/Abstract]
#7 " Lower Limb Dysfunction " [Title/Abstract]
#8 " Extremity Dysfunction " [Title/Abstract]
#9 " Limb " [Title/Abstract]
#10 " Extremity " [Title/Abstract]
#11 #1 OR #2 OR #3 OR #4 OR #5 OR #6 OR #7 OR #8 OR #9 OR #10
#12 " Stoke" [MeSH Terms]
#13 " Stroke* " [Title/Abstract]
#14 " Hemiplegia " [Title/Abstract]

#15 " Cerebrovascular Accident* " [Title/Abstract]

#16 " CVA* (Cerebrovascular Accident) " [Title/Abstract]

#17 " Cerebrovascular Apoplexy " [Title/Abstract]

#18 " Vascular Accident*, Brain" [Title/Abstract]

#19 " Brain Vascular Accident*" [Title/Abstract]

#20 " Cerebrovascular Stroke*" [Title/Abstract]

#21 #12 OR #13 OR #14 OR #15 OR #16 OR #17 OR #18 OR #19 OR #20

#22 " Constraint-Induced Movement Therapy " [MeSH Terms]

#23 " Constraint Induced Movement Therapy " [Title/Abstract]

#24 " CIMT " [Title/Abstract]

#25 " modified Constraint Induced Movement Therapy " [Title/Abstract]

#26 " modified CIMT " [Title/Abstract]

#27 " Constraint Induced Therapy " [Title/Abstract]

#28 " Forced Use Therapy " [Title/Abstract]

#29 #22 OR #23 OR #24 OR #25 OR #26 OR #27 OR #28

#30 #11 AND #21 AND #29

Web of Science search strategy

Search Date: 31st October 2019

#1 TS= ((Movement Disorders) or (Movement Disorder*) or (Dyskinesia Syndrome) or (Motor dysfunction) or (Limb Dysfunction) or (Upper Limb Dysfunction) or (Lower Limb Dysfunction) or (Extremity Dysfunction) or (Limb) or (Extremity))

#2 TS= ((Stroke) or (Stroke*) or (Hemiplegia) or (Cerebrovascular Accident*) or (CVA* (Cerebrovascular Accident)) or (Cerebrovascular Apoplexy) or (Vascular Accident*, Brain) or (Brain Vascular Accident*) or (Cerebrovascular Stroke*))

#3 TS= ((Constraint-Induced Movement Therapy) or (Constraint Induced Movement Therapy) or (CIMT) or (modified Constraint Induced Movement Therapy) or (modified CIMT) or (Constraint Induced Therapy) or (Forced Use Therapy))

#4 #1 AND #2 AND #3
